# Conformal Metamaterials with Active Tunability and Self-Adaptivity for Magnetic Resonance Imaging

**DOI:** 10.34133/research.0560

**Published:** 2024-12-23

**Authors:** Ke Wu, Xia Zhu, Xiaoguang Zhao, Stephan W. Anderson, Xin Zhang

**Affiliations:** ^1^ Department of Mechanical Engineering, Boston University, Boston, MA 02215, USA.; ^2^ Photonics Center, Boston University, Boston, MA 02215, USA.; ^3^ Boston University Chobanian & Avedisian School of Medicine, Boston, MA 02118, USA.

## Abstract

Metamaterials hold great potential to enhance the imaging performance of magnetic resonance imaging (MRI) as auxiliary devices, due to their unique ability to confine and enhance electromagnetic fields. Despite their promise, the current implementation of metamaterials faces obstacles for practical clinical adoption due to several notable limitations, including their bulky and rigid structures, deviations from optimal resonance frequency, and inevitable interference with the radiofrequency (RF) transmission field in MRI. Herein, we address these restrictions by introducing a flexible and smart metamaterial that enhances sensitivity by conforming to patient anatomies while ensuring comfort during MRI procedures. The proposed metamaterial selectively amplifies the magnetic field during the RF reception phase by passively sensing the excitation signal strength, remaining “off” during the RF transmission phase. Additionally, the metamaterial can be readily tuned to achieve a precise frequency match with the MRI system through a controlling circuit. The metamaterial presented here paves the way for the widespread utilization of metamaterials in clinical MRI, thereby translating this promising technology to the MRI bedside.

## Introduction

Metamaterials, rationally designed assemblies of subwavelength unit cells (meta-atoms), enable the unique capacity to tailor the effective properties of artificial materials, thereby realizing characteristics not found in naturally occurring materials, such as left-handed materials or negative refractive index materials [1–3]. With extraordinary electromagnetic (EM) properties, myriad efforts in developing metamaterials have not been limited simply to demonstrating that the metamaterials’ properties extend beyond those of natural materials by breaking generalized limitations of refraction and reflection, but have also been focused on developing novel metamaterial-enabled technologies to facilitate a range of practical applications from the microwave to the optical regime, such as cloaking devices [4], perfect absorbers [5], super lenses [6], among others. One notable property of metamaterials is the near-field enhancement due to their ability to confine the energy of incident radiation to a sub-wavelength region in the vicinity of the metamaterials. When excited by an incident wave at their resonance frequencies, such metamaterials generate an intense and localized electric field at the edges of their narrow capacitive gaps where the induced transient charge accumulates. Such electric field confinement and its corresponding enhancement due to metamaterials has enabled a wide range of applications, including resonant antennas [7,8], plasmon optical tweezers [9], and nonlinear-based devices for phase conjugation [10], to name a few. Just as with the case of electric field confinement, a similar confinement effect also occurs with the magnetic field. At resonance, the induced circulating current distributes along metamaterials’ conductive or metallic traces, leading to a resonant enhancement of the local magnetic field near the regions of the peak transient current. This capacity of magnetic metamaterials for magnetic field enhancement has enabled their application to wireless power transfer [11,12], high-sensitivity sensing [13], and second-harmonic generation [14], among others.

Leveraging their unique capacity for magnetic field confinement and enhancement, metamaterials offer a new perspective on boosting the imaging performance of magnetic resonance imaging (MRI) in a wireless and cost-effective manner [15–17]. When excited by an external radiofrequency (RF) field, the interaction of EM resonators within metamaterials leads to various synergistic resonating modes. Among these modes, there exists a particular mode, in which the induced current along the resonators aligns in the same direction. These synchronized currents collectively produce a secondary magnetic field that augments the primary excitation field, resulting in its amplification. When integrated into MRI systems, the resonant mode of metamaterials can be activated by the magnetic field during the signal reception process. This activation leads to a substantial enhancement in the strength of the MRI signal. Consequently, there is a marked improvement in the signal-to-noise ratio (SNR) of MRI images, facilitating more precise and detailed imaging outcomes. In recent times, a range of metamaterials and metasurfaces, composed of arrays of unit cells with diverse configurations such as split loops, helical coils, coaxially shielded loops, and parallel wires, have been utilized as supplementary devices in MRI to enhance imaging capabilities [18–31]. However, substantial barriers remain to be overcome for their clinical adoptions.

The majority of the reported metamaterials for enhancing MRI systems are typically constructed with bulky and rigid structures, which not only compromise patient comfort but also, importantly, markedly limits the optimized imaging of curved surfaces, such as the brain, neck, or musculoskeletal system (knee, ankle, etc.), as the SNR gains of metamaterials decay rapidly as a function of distance from the metamaterial surface. Crucially, one notable limitation of conventional metamaterials applied to MRI is their intrinsic linearity, which leads to magnetic field enhancement regardless of excitation power strength. This results in field enhancement during both the RF reception and transmission phases. While the enhancement of the RF field B1− during the reception phase results in a desired boost of SNR, the amplification of the RF field B1+ during the transmission phase leads to undesired problems, such as unpredictable deviations in flip angle (FA), suboptimal performance, and potential safety concerns. Nonlinear metamaterials, whose EM behavior is not only dependent on frequency but also affected by the intensity of the incident RF field [32–35], provide a promising method to design self-adaptive metamaterials for MRI. This results in an “off” state during RF transmission and a desired “on” state during RF reception. Despite several investigations aimed at understanding the nonlinear properties of metasurfaces to mitigate interference in RF transmitting fields, they exhibit several drawbacks inherent in their designs. For instance, due to configuration limitations, enlarging the size of metamaterials to ensure compatibility with high-field MRI systems (>1.5 T) proves to be a major challenge [21]. Additionally, in the case of nonlinear metamaterials combined with a nonlinear ring, their self-adaptivity or passive detuning may fail when these metamaterials operate under substantial curvature changes in their configurations [27,30]. Beyond the intrinsic linear response, currently reported metamaterials lack tunability in their resonance frequency. The metamaterial can only amplify the MRI signal to its maximum potential when excited at its resonance frequency. To achieve optimal performance in MRI, it is essential to ensure a precise match between the metamaterial’s resonance frequency and that of the MRI system, thereby maximizing resonant magnetic field enhancement. However, the resonance frequency of metamaterials is susceptible to their local environments and the presence of materials with different permittivities; thus, the metamaterial resonance frequency may shift to undesired values when in proximity to patients with varying body composition (differing degrees of water, fat, muscle, or bone) during an MRI scan. Of note, as with the conformal metamaterial reported herein, the structural deformation may also exert an influence on its resonance frequency by altering the coupling coefficient between unit cells in the metamaterial. Metamaterials incorporating active materials as components of their constitutive elements or integrating structures enabling physical perturbation have been reported to yield the capacity for EM tunability [36–40]. Specifically, for MRI applications, the focus herein, we previously reported a class of mechanically tunable metamaterials inspired by auxetics [23,24]. Though simple and straightforward to implement, manually adjusting these metamaterials poses practical challenges due to issues with precision and efficiency in clinical scenarios.

In this study, we designed a smart metamaterial to enhance MRI signals at a sub-wavelength scale by investigating the underlying mechanisms of nonlinear and tunable metamaterials, grounded in electromagnetism, coupled mode theory, and nonlinear physics. The proposed metamaterial design introduces a novel approach by integrating conventional MRI metamaterials with a control circuit. This integration imparts multiple advanced properties to the metamaterials, enhancing their practicality in clinical MRI applications by addressing challenges such as their inherently linear behavior and resonance frequency deviations. Specifically, this innovative design features an array of meta-atoms, which are composed of voltage-controlled spiral resonators and nonlinear ring resonators. By leveraging the rectifying effect and bistable nonlinear behavior of PN junctions in diodes, the metamaterial’s nonlinearity enables self-adaptive passive detuning during the RF transmission phase, preventing interference with the RF transmission field and ensuring patient safety. The voltage-driven frequency tunability ensures alignment with the working frequency of MRI systems, optimizing the enhancement of the MRI signal. The performance of the metamaterial is thoroughly evaluated through simulation, on-bench measurements, and validations through a clinical MRI system.

## Results

### Modeling of the meta-atom and metamaterial characterization

**Fig. 1.**
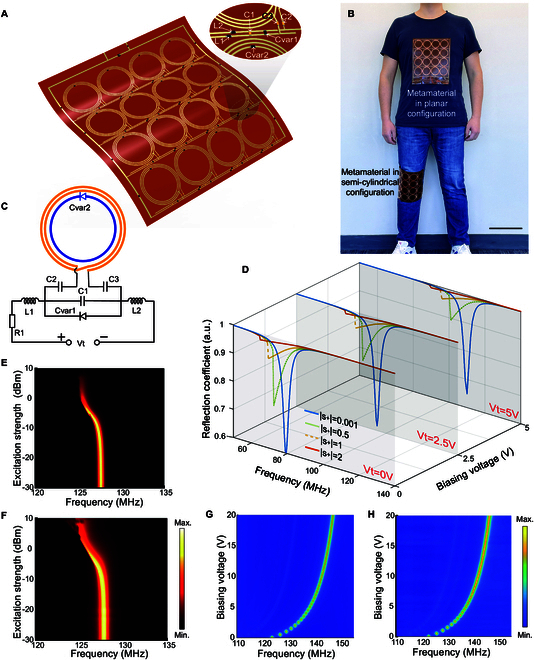
Concept and characterizations of the metamaterial. (A) Image of the metamaterial in a flexible state. (B) Photograph of a human wearing the proposed metamaterial. Scale bar, 20 cm. (C) Schematic diagram of the equivalent circuit of the unit cell in the metamaterial. (D) Theoretical frequency responses of the unit cell. (E and F) Experimentally measured reflection spectra as a function of excitation power strength when the metamaterial is configured in planar (E) and semi-cylindrical shapes (F). (G and H) Experimentally measured reflection spectra of the metamaterial as a function of the biasing voltage when metamaterial is configured in planar (G) and semi-cylindrical shapes (H).

To address the aforementioned general and inherent limitations of conventional metamaterials, we fabricated a conformal metamaterial with frequency tunability and self-adaptivity for MRI applications (Text S1, Fig. S1, and Table S1). The ultra-thin, flexible structure serves to ensure a conformal approximation between the metamaterial and the surface of the object of interest as well as alleviates potential patient discomfort due to inherently rigid, bulky structures. The designed metamaterial consists of a unit cell array arranged in a 4 × 4 fashion, fabricated on an ultra-thin flexible polyimide substrate, as depicted in Fig. 1A and B. The unit cells feature a controlling circuit loaded spiral resonator (CCLSR) inductively coupled with a varactor loaded ring resonator (VLRR), whose equivalent circuit diagram is plotted in Fig. 1C. In the controlling circuit, capacitors *C_2_* and *C_3_* isolate the 2-turn spiral coil from the biasing voltage. Capacitor *C_1_* is used to roughly adjust the resonance frequency of the CCLSR to match the Larmor frequency, while resistor *R_1_* limits the current in the circuit. Since the unit cells in the metamaterial are electrically connected through the biasing voltage lines, inductors *L_1_* and *L_2_* are employed to provide isolation between them. The varactor *C_var1_* offers variable capacitance controlled by the biasing voltage *V_t_*. The resonance frequency of the CCLSR may be expressed as $\omega =1/\sqrt{\mathrm{LC}}$ , in which the inductance *L* is predominantly attributed to the inductance of the spiral coil, while the effective capacitance *C* may be expressed by:

CCCLSR=Cs+11C1+Cvar1Vt+1C2+1C3=Cs+11C1+C01+VtVP−M+CP+1C2+1C3 (1)

in which *C_s_* is the distributed capacitance in the spiral coil, *C_0_* is the initial capacitance of varactor *C_var1_*, *M* is a fitting exponent, and *V_P_* is the intrinsic potential of the varactor obtained from the data sheet. *V_t_* is the voltage across the varactor applied by the controlling circuit. As for the VLRR component, its effective inductance mainly results from the inductance of the ring loop, with its capacitance being expressed by [27,32]:

$$C_{\text{VLRR}}=C_{\mathrm{var}2}\left(V_{D}\right)=C_{0}{\left(1-\frac{V_{D}}{V_{P}}\right)}^{-M}$$ (2)

in which *V_D_* is the voltage across the varactor *C_var2_*. Unlike the driving voltage *V_t_* across the varactor *C_var1_*, which could be adjusted at will by the controlling circuit, the voltage across the varactor *C_var2_* results from the rectifying effect of the diode junction when excited by an RF wave. Consequently, the discrepancy in excitation field strength will automatically trigger the adaptive behavior. When excited by an RF wave with relatively low power, the induced voltage *V_D_* is much smaller than *V_P_*, resulting in an effective capacitance of the VLRR close to the initial capacitance of the varactor *C_0_*. However, when excited by an RF wave of high power, the oscillation strength of the VLRR becomes stronger, inducing a higher driving voltage across the varactor *C_var2_*, which, in turn, increases the capacitance of the varactor, thereby shifting the resonance frequency to a lower value. Simultaneously, the bistable nonlinear behavior in the amplitude response will result in an attenuation of the oscillating strength when the excitation strength reaches a sufficiently high level. This excitation-dependent response endows the VLRR with the desired self-adaptivity to the power strength of the incident excitation. Of note, the integrated electronic components employed in the metamaterial will introduce series resistance, which inevitably introduces loss and reduces the *Q* value of the metamaterial. To achieve the optimal performance of the metamaterial, we investigated and evaluated the impact of the series inner resistance on the SNR enhancement performance in Text S2, Fig. S2, and Table S2. When these 2 resonators, i.e., the tunable CCLSR and the self-adaptive VLRR, are placed in close proximity to one another, they strongly interact with one another, which may be expressed by the coupling factor *k*. Their resonance response may be mathematically described using the coupled-mode theory (CMT) [41,42]. Assuming that the external excitation signal is a harmonic function with frequency ω (i.e., s_+_ = |s_+_|e^jωt^), the oscillating amplitude of the 2 resonators may be calculated by numerically solving the matrix equations:

jωa1a2=jω1Vt+j1τe1+1τo1kkω2a2+j1τe2+1τo2a1a2+2τe12τe2s+ (3)

In the expression, the subscripts “1” and “2” denote the resonator CCLSR and the VLRR, respectively. *a_n_* (where *n* = 1 or 2) is the mode amplitude of the resonator, *ω_n_* represents the self-resonance frequency, and *1/τ_en_* and *1/τ_0n_* are the decay rates due to radiation and intrinsic losses, respectively. *s+* is the excitation signal, and $\sqrt{2/\tau_{\mathrm{en}}}$ is the coefficient representing the degree of coupling between the resonator and the excitation wave. *ω_1_* is a function of the biasing voltage *V_t_* applied in the CCLSR, and *ω_2_* depends on the oscillation strength of the VLRR. Consequently, the overall response of the unit cell (when considering the CCLSR and VLRR components as a whole) is not only modulated by the biasing voltage *V_t_*, but also dependent on the excitation power strength. The reflection coefficient of the unit cell may be expressed by [41]:

$$r=-1+\frac{\sqrt{\frac{2}{\tau_{e1}}}a_{1}+\sqrt{\frac{2}{\tau_{e2}}}a_{2}}{2\left|s_{+}\right|}$$ (4)

The reflection spectra as a function of incident wave *s_+_* with varying power levels and biasing voltages *V_t_* are plotted in Fig. 1D. Details of the calculations can be found in Text S3 and Table S3. The dips in the curves indicate the resonance frequency and the oscillation strength of the unit cell. At a given biasing voltage (e.g., *V_t_* = 0 V), when the excitation power strength is low (e.g., |*s_+_*| = 0.001), the unit cell resonates at the designated resonance frequency with a strong oscillation amplitude, which yields a marked magnetic field enhancement in the vicinity of the unit cell. As the excitation power increases, the resonance of the unit cell shifts to a lower frequency. Additionally, the peak oscillation amplitude at the resonance frequency decreases along with the frequency shift, which results from the bistable nonlinear behavior in the amplitude response of the VLRR [32]. The attenuation of the peak amplitude resulting from the strong excitation field ensures that the unit cell will not enhance the magnetic field and will not interfere with the incident RF wave. Another notable property of the unit cell is the frequency tunability introduced by the controlling circuit. When the biasing voltage is adjusted to 2.5 or 5 V, as shown in Fig. 1D, the resonance frequency shifts to a higher value, and this variation in biasing voltage does not impact the self-adaptive response to the incident power. In this manner, the resonance frequency can be precisely tuned to ensure a frequency match without disrupting the self-adaptive response to the excitation power strength.

With the aforementioned fundamental mechanism of the unit cells within the metamaterial, we sought to experimentally validate the resultant advanced properties through the assembly of the meta-atoms. Initially, the metamaterial was configured in a planar shape (Fig. S3A), with the resonance frequency tuned to ~127 MHz. The self-adaptive response to the excitation power strength was verified using a network analyzer with adjustable excitation power. The reflection spectra *S11* were measured across a range of excitation power strengths, with the results plotted in Fig. 1E. Initially, the resonance frequency of the metamaterial remained constant at ~127 MHz, with minimal change in oscillation amplitude for excitation power level within −8 dBm. As the excitation power strength increased, the resonance frequency shifted to a lower value, accompanied by an attenuation of the oscillation amplitude. When the excitation power strength reached a sufficiently high level (e.g., >7 dBm), an abrupt transition in the spectrum occurred, and oscillation in the metamaterial ceased. In the case of MRI, the RF power during the reception phase is in the μW range, while the power during the transmission phase reaches the kW range [43]. This marked difference in power levels between the reception and transmission phases in MRI is sufficiently large to cause the metamaterial to exhibit its self-adaptive response as a function of excitation power strength. A comparative schematic illustration of the proposed metamaterial and a conventional nonadaptive metamaterial, showing how they interact with the transmission and reception fields in an MRI system, is depicted in Fig. S4. Next, in an effort to mirror more optimal geometries for imaging nonplanar portions of the human anatomy (wrist, knee, or neck, for example) in a conformal fashion, we measured the *S11* of the curved, semi-cylindrical metamaterial (Fig. S3B). In this case, as plotted in Fig. 1F, an identical response was observed, indicating that the deformation of the metamaterial has no impact on its self-adaptivity. Compared to previous methods that achieved self-adaptivity by integrating a linear metamaterial with a nonlinear ring, introducing nonlinearity to every unit cell within the metamaterial ensures robust self-adaptivity and effective passive detuning. This approach remains effective under large deformations of the metamaterial and allows for arbitrary extensions of the number of unit cells, enabling diverse configurations. Besides the nonlinearity of the metamaterial, the *S11* were measured using a sweep of the biasing voltage to verify its frequency tunability. As the applied biasing voltage changes, the effective capacitance of the varactor *C_var1_* adjusts accordingly. Since the metamaterial’s resonance frequency depends on the effective capacitance of this varactor, the resonance frequency can be actively tuned by controlling the applied biasing voltage. From the experimental results plotted in Fig. 1G, as the biasing voltage increases from 0 to 20 V, the resonance frequency of the metamaterial could be adjusted from 123.5 to 147.0 MHz. Subsequently, a similar experiment was performed with the metamaterial in its semi-cylindrical configuration, with the test results plotted in Fig. 1H. As shown, a resonance frequency tuning range (122.8 to 146.5 MHz) is achieved with the adjustment of the biasing voltage from 0 to 20 V, demonstrating that the curvature of the metamaterial has a negligible effect on its frequency tuning range. Importantly, the ~23-MHz frequency tuning range is sufficient to compensate for detuning effects arising from load variations or structural deformations, which lead to frequency shifts of less than 1.5 MHz (Text S4 and Fig. S5). In comparison to the existing mechanical frequency tunability of previously reported metamaterials, the voltage-driven tunability demonstrates superior precision and responsiveness, allowing for more refined control of the resonance frequency, enabling a simplified, faster workflow while maintaining excellent image quality. These EM characterizations for the metamaterial in different configurations further validate its advanced properties for real-world clinical MRI applications.

### Magnetic field mapping

**Fig. 2.**
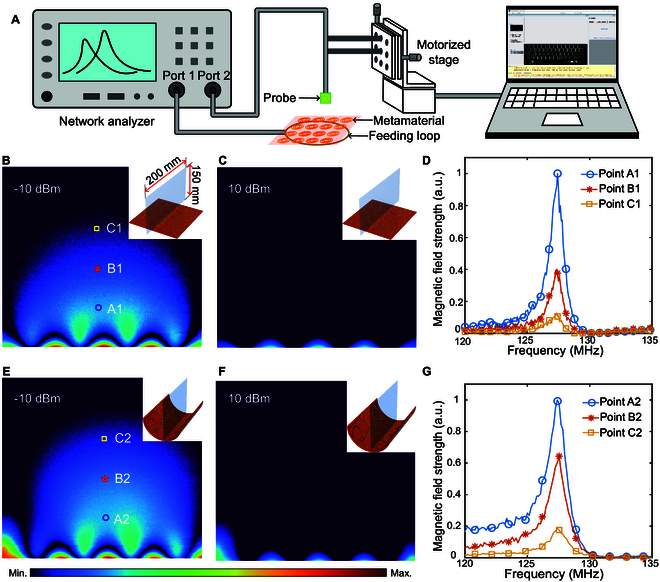
Magnetic field mapping in the vicinity of the metamaterial. (A) Diagram of the field mapping setup. (B and C) Experimentally measured magnetic field strength distributed along the planar metamaterial cross-section (depicted as the blue plane in the inset) at different excitation strengths, e.g., *s_+_* = −10 dBm (B) and *s_+_* = 10 dBm (C). (D) Spectra of the magnetic field distribution at points A1, B1, and C1, as shown in (B). (E and F) Magnetic field strength distributed along the semi-cylindrical metamaterial cross-section (depicted as the blue plane in the inset) at different excitation strengths, e.g., *s_+_* = −10 dBm (E) and *s_+_* = 10 dBm (F). (G) Spectra of the magnetic field distribution at points A2, B2, and C2, as shown in (E).

To experimentally demonstrate magnetic field confinement and enhancement by the metamaterial, a near-field mapping setup was built to measure the magnetic field patterns in the space (Fig. 2A and Fig. S6). First, the planar metamaterial was excited by an RF power of −10 dBm. The normalized magnetic field distribution on the cutting plane (indicated by the blue plane shown in the inset of Fig. 2B) was measured and depicted in Fig. 2B. In the vicinity of the metamaterial, the resonant oscillating currents within the unit cells collectively boost the original excitation magnetic field. This enhanced magnetic field pattern identifies the optimal region for placing samples to be imaged in MRI. Using the same experimental setup, the magnetic field was also mapped under a higher excitation power of 10 dBm, as shown in Fig. 2C. In contrast, the magnetic field strength approximates 0, validating that the metamaterial effectively turned “off” under high power excitation. The absence of an appreciable magnetic field in this case further demonstrates its self-adaptive response to the transmitting field B1+ when applied to MRI. In addition, the spectra of the magnetic field strength at 3 distinct locations 30, 70, and 110 mm away from the metamaterial surface (labeled as points A1, B1, and C1 in Fig. 2B) are plotted in Fig. 2D. The magnetic field is maximized at the resonance frequency regardless of the measured location. However, any shift from the resonance frequency substantially reduces the magnetic field strength. For instance, a 2-MHz shift from the resonance frequency results in a 70% decrease in field strength, highlighting the importance of precise frequency matching between the metamaterial and the MRI system. Next, magnetic field distributions were mapped for the metamaterial configured in a semi-cylindrical shape. The magnetic field patterns under excitation powers of −10 and 10 dBm are plotted in Fig. 2E and F, respectively, and the spectra of the magnetic field strength at the same locations are presented in Fig. 2G. Similar conclusions may be drawn from these results for the semi-cylindrical metamaterial. Furthermore, by comparing the spectra of the magnetic field strength in Fig. 2D and G, it is evident that the semi-cylindrical configuration of the metamaterial has a notable advantage: The field strength decays more slowly than with the planar metamaterial, resulting in a greater penetration depth of the field enhancement. This increased penetration depth has important practical implications for clinical MRI, where imaging deeper anatomical structures may be of interest. In addition to the experimental results, we also built a numerical simulation model to investigate the magnetic field distribution, including both magnitude and phase information (Text S5 and Figs. S7 and S8). The experimental near-field mapping results closely align with the simulated magnetic field patterns, confirming the accuracy of the experimental findings. Moreover, the simulated phase information of the magnetic field in regions distant from the metamaterial indicates that there are no phase transition regions where the original and induced magnetic fields cancel each other. This prevents RF artifacts in the imaging area due to magnetic field cancellation when the metamaterial is applied to the MRI system.

### MRI validations for metamaterial when combined with the whole body coil

**Fig. 3.**
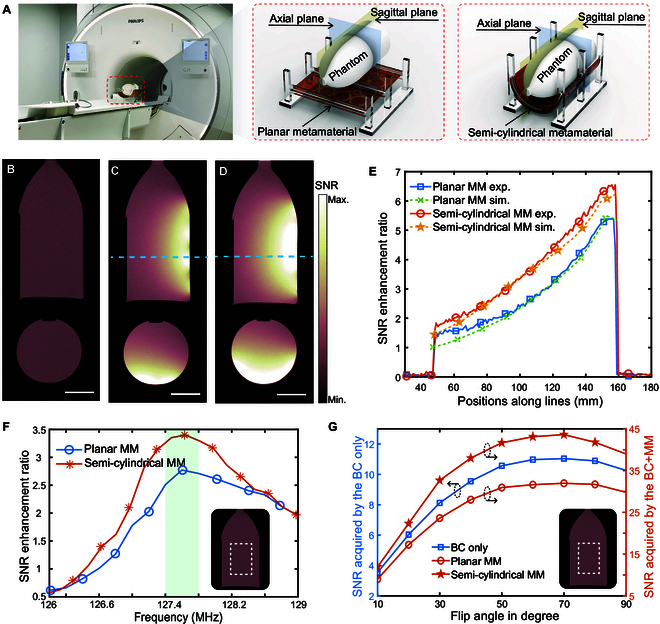
MRI validations for metamaterial when combined with the BC. (A) Experimental setups for metamaterial in planar and semi-cylindrical configurations. (B) SNR images on sagittal and axial planes captured by the BC only. (C) SNR images captured by the BC enhanced by the planar metamaterial. (D) SNR images captured by the BC enhanced by the semi-cylindrical metamaterial. (E) Comparison of the SNR enhancement ratio along the blue dashed lines in (C) and (D). (F) SNR performance as a function of frequency. (G) Variations of the SNR as a function of FA. Scale bars, 5 cm (B to D).

To evaluate the performance of this metamaterial when incorporating with the body coil (BC) in MRI, experimental validations of the SNR enhancement were conducted using a clinical 3T MRI system (Philips Healthcare), as shown in Fig. 3A. The SNR evaluations were performed using the 2-image method (Fig. S9) [18,23,29,31,44], which adheres to the standards set by the National Electrical Manufacturers Association (NEMA) for SNR determination in diagnostic MRI [45]. To validate this approach, we employed another 2 alternative SNR assessment methods recommended by NEMA. The close agreement among these 3 distinct methods confirms the reliability of the 2-image method (Text S6 and Fig. S10). A bottle-shaped phantom filled with mineral oil was initially scanned by the BC in the absence of the metamaterial, which was completely removed from the MRI scanner, serving as a reference standard. The SNR image in the sagittal and axial planes (indicated as the rectangular cutting plane in yellow and the square cutting plane in blue in Fig. 3A) are depicted in Fig. 3B. Next, the phantom was placed on top of the finely tuned metamaterial, with a separation distance of approximately 20 mm. Using the same imaging sequences as the reference, the phantom was scanned by the BC with the planar and semi-cylindrical metamaterials, and the corresponding SNR images are depicted in Fig. 3C and D, respectively. In contrast to the uniform pattern observed in the reference image, the SNR images enhanced by the metamaterials exhibit patterns similar to those in the near-field mapping results in Fig. 2B and E. This further supports the direct correlation between the degree of magnetic field enhancement and resulting SNR enhancement during image acquisition. Moreover, the SNR enhanced by the metamaterials along the dashed lines in the image (shown in Fig. 3C and D) were extracted and normalized to the reference, as plotted in Fig. 3E. This analysis reveals a substantial overall SNR enhancement, up to 6.5-fold compared to the acquisition without the metamaterial. The SNR enhancement ratio by the semi-cylindrical metamaterial was higher at its maximum and decayed at a slower rate compared to the planar configuration, due to the superimposition effects noted above. The mechanism of SNR enhancement by the metamaterial was further explored by deriving the governing equation for SNR, originally formulated by [46]:

$$\text{SNR}\propto \frac{\omega^{2}B_{c}}{\sqrt{R_{\mathrm{BC}}+R_{\text{sample}}+R_{\mathrm{mm}}}}$$ (5)

in which *B_c_* is the magnetic field strength generated with unit current in the receiving coil and *ω* is the Larmor frequency. According to Eq. 5, there are 3 principal dissipative elements contributing to noise sources in MRI. The first is the series resistance of the receive coil *R_BC_*, which results from conductive loss in the receive coil. The second is the equivalent series resistor *R_sample_*, representing power loss in the sample or patient due to induced eddy currents. The third is the equivalent series resistance *R_mm_*, which accounts for conductive power dissipation in the metamaterial. Since the Larmor frequency *ω* is directly proportional to the static field strength *B_0_*, it is a constant value for the experimental setup described here. Thus, the analytical SNR enhancement ratio could be calculated by dividing the simulated magnetic field strength by the square root of the total power dissipation in the BC, metamaterial, and phantom, with all these parameters readily to be extracted from the simulation results (Text S7 and Fig. S11). The analytical results, plotted in Fig. 3E, show a high degree of agreement with the MRI experimental results.

To obtain a comprehensive understanding of the correlation between metamaterial enhancement performance and its resonance frequency, we conducted a series of scans of the phantom across a range of resonance frequencies, spanning from 126 to 129 MHz with increments of 0.25 MHz. We extracted the mean SNR values from the outlined regions (as depicted in the inset figure of Fig. 3F) and plotted them as a function of resonance frequency in Fig. 3F. Based on the curve, it can be inferred that the optimal resonance frequency for the metamaterials lies at approximately 127.6 MHz. The optimal frequency for the proposed metamaterials slightly offsets from the Larmor frequency in the 3T MRI system, primarily due to the coupling phenomenon between the metamaterial and the BC. Additionally, for conventional metamaterials without self-adaptivity, their introduction would enhance not only the RF reception field B1− but also transmission field B1+, potentially causing marked image artifacts or even imaging failure. Consequently, to counteract the enhancement of B1+ caused by conventional metamaterials, the transmission energy is generally reduced by decreasing the FA accordingly. To demonstrate the superiority of this self-adaptive metamaterial in this work, another set of MRI validations was performed by imaging the phantom with a sweep in FA from 10° to 90°. The mean value of SNR in the area of interest (outlined by the dashed white rectangle shown in the inset figure of Fig. 3G) as a function of FA is plotted in Fig. 3G. The SNR variations with the sweep of FAs exhibit the same trend, regardless of the absence or presence of the metamaterial. The results further demonstrate that the metamaterial shows an excellent passive detuning and self-adaptive response to B1+ and B1− during the transmission and reception phases.

**Fig. 4.**
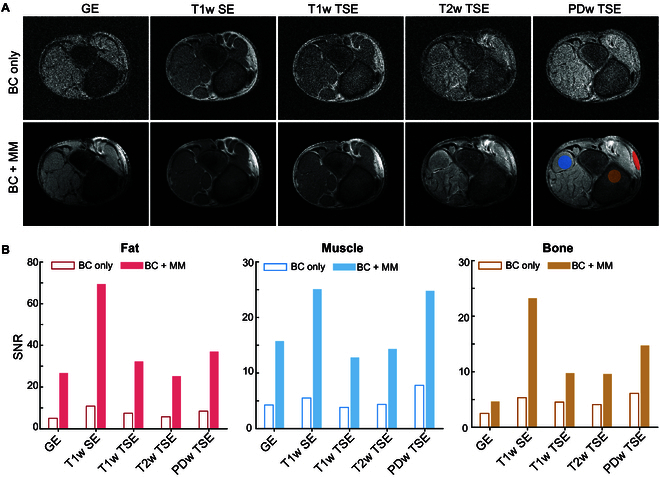
MRI scans of ex vivo porcine leg by the BC through different imaging sequences. (A) SNR images captured with and without the presence of metamaterial. (B) Quantitative assessment of the SNR performance of specific tissues.

In addition to the mineral oil phantom, an ex vivo porcine leg sample was employed in the experimental MRI validations with the BC to preliminarily demonstrate the performance of the metamaterial in biomedically relevant imaging. As opposed to the gradient echo (GE) imaging employed for the mineral oil phantom, T1-weighted turbo spin echo (T1w TSE) imaging featuring a series of 180° refocusing pulses following a single 90° excitations pulse was used for this validation. The porcine leg was wrapped with the metamaterial (Fig. S12), and 8 slices (images) on the axial plane were scanned in the absence and presence of the metamaterial (Fig. S13). These images provide a preliminary demonstration of the potential of the metamaterial to enhance SNR in complex anatomical settings. From the multiple slices obtained with T1w TSE, we selected the cutting plane position for slice #5 in Fig. S13 and conducted scans on the same plane using 4 mainstay pulse sequences commonly used in clinical MRI: GE, T1-weighted spin echo (T1w SE), T2-weighted turbo spin echo (T2w TSE), and proton density-weighted turbo spin echo (PDw TSE). The slices (images) scanned in the absence and presence of metamaterials are depicted in Fig. 4A. Notably, compared to images acquired using the BC only, images with enhanced SNR were achieved in the presence of the metamaterial, although different tissues (fat, muscle, bones, and bone marrow) were included in the leg. Additionally, the result proves that the metamaterial is readily compatible with a variety of clinical RF pulse sequences frequently used in clinical MRI. For quantitative analysis of SNR enhancement in specific tissues, we plotted bar graphs of SNR mean values for muscle, fat, and bone in Fig. 4B. The results reveal substantial SNR gains attributed to the introduction of the metamaterial. Moreover, a larger biological sample, a pineapple, was scanned with the metamaterial in an arc shape between the planar and semi-cylindrical shapes (Text S8 and Fig. S14), further highlighting the potential utility of metamaterial-enhanced MRI.

### MRI validations for metamaterial when combined with the surface coil

**Fig. 5.**
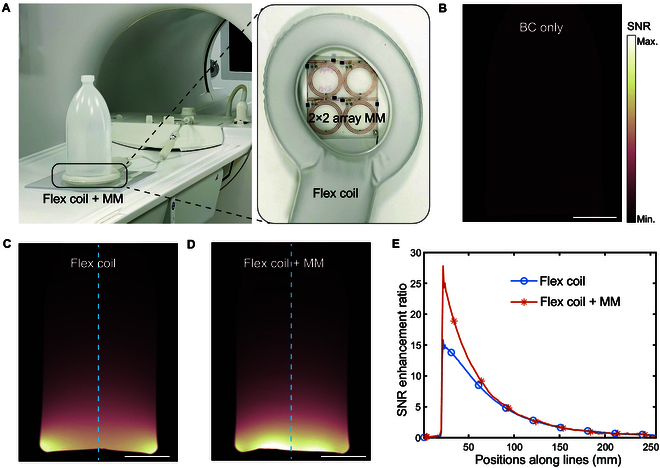
MRI validations for the surface coil enhanced by the metamaterial. (A) Experimental setups. Inset: Configuration of surface coil integrated with the metamaterial. (B) SNR images captured by the BC only. (C) SNR images captured by the surface coil only. (D) SNR image captured by the surface coil combined with the metamaterial. (E) SNR enhancement ratio along blue dashed lines in (C) and (D). Scale bars, 5 cm (B to D).

In this work, our primary objective was to design an advanced metamaterial to enhance the SNR of MRI as an auxiliary device. The metamaterial is not intended to replace current commercial surface coils but rather to augment the imaging capabilities of existing receive coils, including both the BC and surface coils, through their integration. We have demonstrated that the metamaterial may be combined with the BC to boost the imaging power of MRI. More importantly, the metamaterial presented here has the potential to be integrated with surface coils to further enhance their imaging capabilities. To this end, we investigated the performance of the metamaterial when incorporating with the commercial surface coils. We constructed a metamaterial in a 2 × 2 array and integrated it into the dStream Flex M surface coil (Philips Healthcare), as shown in Fig. 5A. Initially, the phantom was scanned separately using the BC and the Flex surface coil only, without the integration of the metamaterial, as shown in Fig. 5B and C. Subsequently, we conducted another scan using the Flex surface coil with the metamaterial integrated. The resulting SNR image is presented in Fig. 5D. For quantitative comparisons, we extracted the SNR enhancement ratio along dashed lines in Fig. 5C and D by normalizing to the BC-only image, as shown in Fig. 5E. The switch from the BC to the Flex coil resulted in a dramatic improvement in SNR. Specifically, there was an approximately 15-fold increase in SNR at the bottom of the phantom near the Flex coil. More importantly, the integration of the metamaterial into the Flex coil led to a nearly 2-fold increase in SNR compared to the Flex coil only image. These MRI validations demonstrate that metamaterials have the potential to not only enhance the imaging capabilities of the BC but also substantially improve the performance of surface coils. This opens up new possibilities for the application of metamaterials in MRI across a wide range of scenarios.

**Fig. 6.**
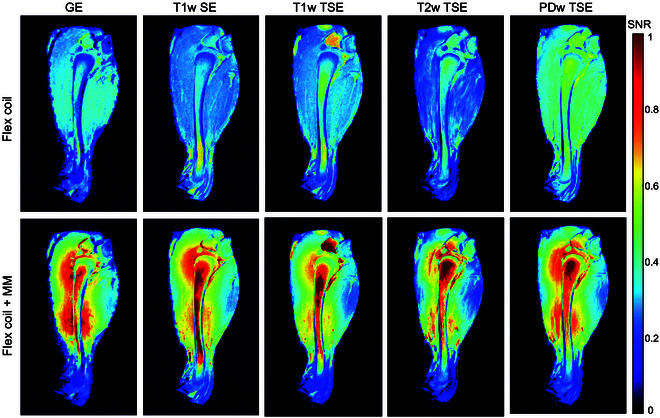
Comparisons between images captured by the Flex coil in the absence and presence of the metamaterial.

Finally, we sought to validate the performance of surface coil enhanced by the metamaterial with an ex vivo chicken leg. We placed the chicken leg on top of the Flex coil and employed the same scanning sequences previously used with the porcine leg. SNR maps of the chicken leg were generated by normalizing to the background noise level, as shown in Fig. 6. By comparing the images acquired using the Flex coil with and without the integrated metamaterial, we observed that the metamaterial has the potential to enhance not only the performance of the BC but also that of surface coils. This suggests its applicability for improving the imaging capabilities of MRI across various clinical RF transmission pulse sequences commonly used in MRI.

## Discussion

This work demonstrates a conformal, intelligent metamaterial capable of selectively boosting the B1− field under optimized frequency-matched conditions and self-adaptively switching to an off-resonance state without interfering with the B1+ field. This results in marked improvements in SNR enhancement performance when used in MRI. In contrast to conventional metamaterials, the meta-atom of the reported metamaterial integrates a tunable CCLSR and a nonlinear VLRR, which provide voltage-driven frequency tunability and RF field-dependent resonance response. These advanced features allow the metamaterial to enhance both the BC and surface coils, highlighting its potential for widespread use in MRI systems. Attributed to these advanced features, the metamaterial has the potential to serve as an auxiliary device, enhancing not only the imaging capabilities of the BC but also those of surface coils, exhibiting great promise for widespread utilization within MRI systems. The mathematical modeling based on CMT and subsequent bench characterizations elucidate the metamaterial’s governing physical mechanism, offering a deep analysis of its frequency-tunable and field-dependent resonant behavior. This metamaterial reported here is adaptable to different 3T MRI systems regardless of the manufacturer. Furthermore, the metamaterial is readily adjusted to be applicable to MRI system with different static magnetic field strengths, such as 1.5, 7.0, or 9.4 T. Since these superior properties of metamaterials are predominately derived from their constituent meta-atoms, the metamaterial remains valid with varying numbers of unit cells, allowing for customization into different shapes and configurations for specific applications. Ultimately, the voltage-driven frequency tuning mechanism and field-dependent resonance response offer a promising approach for future EM devices aimed at near-field manipulation, extending beyond MRI applications. Future efforts could focus on addressing the challenges posed by metamaterials on certain postprocessing features of the MR scanner’s reconstruction pipeline by precisely mapping metamaterial sensitivity and adjusting postprocessing algorithms accordingly. Another direction for future work, considering clinical implementation, involves specific absorption rate (SAR) analysis through numerical simulations and the development of experimental setups for monitoring temperature variations during in vivo scans.

## Materials and Methods

### Geometry and fabrication of the metamaterial

The reported metamaterial was fabricated using 0.089-mm-thick copper foil tape, supported on a flexible polyimide film (Kapton) dielectric layer with dimensions of 305 × 305 mm and a thickness of 0.013 mm. The CCLSR had an inner radius of 20.4 mm, a strip width of 0.8 mm, and an inter-strip spacing of 0.8 mm. The VLRR, sharing the same concentric point with the CCLSR, had an inner radius of 18.8 mm and a strip width of 0.8 mm. The metamaterial was realized by assembling 4 × 4 and 2 × 2 arrays, with the column separation distance of neighboring unit cells being 45.6 mm, and the row separation distance being 48.8 mm. Biasing voltage lines were inserted between the neighboring rows for applying biasing voltage to the controlling circuits of each unit cell.

### Characterization of the metamaterial

A vector network analyzer (VNA; E5071C, Keysight Inc.) with a feeding loop was employed to excite the magnetic resonance of the metamaterial. To precisely measure the resonance frequency of the metamaterial, we moved the feeding loop from a distance and gradually approached the metamaterial. When the reflection spectrum began to show a dip, we stabilized the feeding loop to record the spectrum. This method avoids excessive coupling between the feeding loop and the metamaterial, as over-coupling could slightly shift the self-resonance frequency. The reflection spectra were measured with a biasing voltage sweep from 0 to 20 V in steps of 0.5 V. For characterizing the metamaterial’s self-adaptivity to excitation power strength, the biasing voltage was adjusted to tune the resonance frequency to ~127 MHz. The reflection spectra were measured with an excitation power sweep from −30 to 10 dBm in steps of 1 dBm.

### Numerical simulation

Numerical simulations were performed using CST Microwave Studio software. The simulation model’s dimensions were identical to the fabricated sample described above. The mineral oil phantom was modeled with a relative permittivity of 2.1, electric conductivity of 0.175 S/m, and material density of 800 kg/m^3^.

### Magnetic field mapping

In this setup, a feeding loop with a diameter of 250 mm was connected to port 1 of the VNA. The RF signal fed into the coupling loop served as the excitation wave. A small circular loop with a diameter of 10 mm served as the probe, connected to port 2 of the VNA via a coaxial cable. The judiciously designed probe size was small enough to minimize its impact on the field distribution of the metamaterial, ensuring a high spatial resolution of the magnetic field mapping. Simultaneously, it was large enough to detect the magnetic field near the metamaterial when excited by low power. The probe was mounted on a 2-dimensional motorized stage controlled by a computer, allowing for data collection across a wide array of locations surrounding the metamaterial. Measurements were taken at 200 × 150 data points over an area of 200 × 150 mm. The transmission coefficient (S21) was measured to indicate the magnetic field strength.

### MRI validation

For MRI validations with a phantom, a GE imaging sequence was employed using a repetition time (TR) of 100 ms and an echo time (TE) of 4.6 ms. GE imaging was first performed to capture a phantom image (Fig. S9A), followed by capturing a noise image by shutting down the transmission RF coil (Fig. S9B). The SNR images of the phantom were calculated by taking the ratio between the mean value of the magnitude phantom image and the standard deviation of the noise image. The ex vivo samples of porcine and chicken legs used in this work were obtained from a local butcher shop.

## Data Availability

All data needed to evaluate the conclusions in the paper are present in the paper and/or the Supplementary Materials.

## Supplementary Materials

Supplementary 1 Texts S1 to S8 Figs. S1 to S14 Tables S1 to S3

## References

[1] Pendry JB, Holden AJ, Robbins DJ, Stewart WJ. Magnetism from conductors, and enhanced non-linear phenomena. IEEE Trans Microw Theory Techniq. 1999;47(11):2075–2084.

[2] Smith DR, Padilla WJ, Vier DC, Nemat-Nasser SC, Schultz S. Composite medium with simultaneously negative permeability and permittivity. Phys Rev Lett. 2000;84:4184–4187. 10990641 10.1103/PhysRevLett.84.4184

[3] Pendry JB. Negative refraction makes a perfect lens. Phys Rev Lett. 2000;85:3966–3969. 11041972 10.1103/PhysRevLett.85.3966

[4] Schurig D, Mock JJ, Justice B, Cummer SA, Pendry JB, Starr AF, Smith DR. Metamaterial electromagnetic cloak at microwave frequencies. Science. 2006;314(5801):977–980. 17053110 10.1126/science.1133628

[5] Landy NI, Sajuyigbe S, Mock JJ, Smith DR, Padilla WJ. Perfect metamaterial absorber. Phys Rev Lett. 2008;100:Article 207402. 18518577 10.1103/PhysRevLett.100.207402

[6] Zhang X, Liu ZW. Superlenses to overcome the diffraction limit. Nat Mater. 2008;7(6):435–441. 18497850 10.1038/nmat2141

[7] Dong Y, Itoh T. Metamaterial-based antennas. Proc IEEE. 2012;100(7):2271–2285.

[8] Mühlschlegel P, Eisler HJ, Martin OJF, Hecht B, Pohl DW. Resonant optical antennas. Science. 2005;308(5728):1607–1609. 15947182 10.1126/science.1111886

[9] Juan ML, Righini M, Quidant R. Plasmon nano-optical tweezers. Nat Photonics. 2011;5(6):349–356.

[10] Katko AR, Gu S, Barrett JP, Popa BI, Shvets G, Cummer SA. Phase conjugation and negative refraction using nonlinear active metamaterials. Phys Rev Lett. 2010;105:Article 123905. 20867644 10.1103/PhysRevLett.105.123905

[11] Wang B, Yerazunis W, Teo KH. Wireless power transfer: Metamaterials and array of coupled resonators. Proc IEEE. 2013;101(6):1359–1368.

[12] Wu K, Duan G, Zhao X, Chen C, Anderson SW, Zhang X. Metamaterial-enhanced near-field readout platform for passive microsensor tags. Microsyst Nanoeng. 2022;8:28. 35310512 10.1038/s41378-022-00356-4 PMC8891326

[13] Chen J, Nie H, Peng C, Qi S, Tang C, Zhang Y, Wang L, Park GS. Enhancing the magnetic plasmon resonance of three-dimensional optical metamaterials via strong coupling for high-sensitivity sensing. J Lightwave Technol. 2018;36(16):3481–3485.

[14] Klein MW, Enkrich C, Wegener M, Linden S. Second-harmonic generation from magnetic meta-materials. Science. 2006;313(5786):502–504. 16873661 10.1126/science.1129198

[15] Wiltshire MCK, Pendry JB, Young IR, Larkman DJ, Gilderdale DJ, Hajnal JV. Microstructured magnetic materials for RF flux guides in magnetic resonance imaging. Science. 2001;291(5505):849–851. 11157159 10.1126/science.291.5505.849

[16] Freire MJ, Marques R, Jelinek L. Experimental demonstration of a μ=− 1 metamaterial lens for magnetic resonance imaging. Appl Phys Lett. 2008;93:231108.

[17] Dale BM, Brown MA, Semelka RC. *MRI: Basic principles and applications*. Chichester: John Wiley & Sons Inc.; 2015.

[18] Slobozhanyuk AP, Poddubny AN, Raaijmakers AJ, van Den Berg CA, Kozachenko AV, Dubrovina IA, Melchakova IV, Kivshar YS, Belov PA. Enhancement of magnetic resonance imaging with metasurfaces. Adv Mater. 2016;28(9):1832–1838. 26754827 10.1002/adma.201504270

[19] Shchelokova AV, van den Berg CA, Dobrykh DA, Glybovski SB, Zubkov MA, Brui EA, Dmitriev DS, Kozachenko AV, Efimtcev AY, Sokolov AV, et al. Volumetric wireless coil based on periodically coupled split-loop resonators for clinical wrist imaging. Magn Reson Med. 2018;80(4):1726–1737. 29427296 10.1002/mrm.27140

[20] Duan G, Zhao X, Anderson SW, Zhang X. Boosting magnetic resonance imaging signal-to-noise ratio using magnetic metamaterials. Commun Phys. 2019;2(1):35. 31673637 10.1038/s42005-019-0135-7 PMC6822984

[21] Chi Z, Yi Y, Wang Y, Wu M, Wang L, Zhao X, Meng Y, Zheng Z, Zhao Q, Zhou J. Adaptive cylindrical wireless metasurfaces in clinical magnetic resonance imaging. Adv Mater. 2021;33(40):2102469. 10.1002/adma.202102469 34402556

[22] Slobozhanyuk AP, Shchelokova AV, Dobrykh DA, Seregin PS, Powell DA, Shadrivov IV, Webb AG, Belov PA, Lapine M. Detunable wire metasurface for applications in magnetic resonance imaging. Bull Russ Acad Sci Phys. 2022;86:S216–S221.

[23] Wu K, Zhao X, Bifano TG, Anderson SW, Zhang X. Auxetics-inspired tunable metamaterials for magnetic resonance imaging. Adv Mater. 2022;34(6):2109032. 10.1002/adma.202109032 PMC8831474 34865253

[24] Wu K, Zhu X, Bifano TG, Anderson SW, Zhang X. Computational-design enabled wearable and tunable metamaterials via freeform auxetics for magnetic resonance imaging. Adv Sci. 2024;11(26):e2400261. 10.1002/advs.202400261 PMC11234395 38659228

[25] Das P, Gupta J, Sikdar D, Bhattacharjee R. A thin metallo-dielectric stacked metamaterial as “add-on” for magnetic field enhancement in clinical MRI. J Appl Phys. 2022;132:114901.

[26] Gupta J, Das P, Bhattacharjee R, Sikdar D. Enhancing signal-to-noise ratio of clinical 1.5 T MRI using metasurface-inspired flexible wraps. Appl Phys A. 2023;129:725.

[27] Zhao X, Duan G, Wu K, Anderson SW, Zhang X. Intelligent metamaterials based on nonlinearity for magnetic resonance imaging. Adv Mater. 2019;31(49):1905461. 10.1002/adma.201905461 PMC7108751 31663651

[28] Zhu X, Wu K, Anderson SW, Zhang X. Helmholtz coil-inspired volumetric wireless resonator for magnetic resonance imaging. Adv Mater Technol. 2023;8(22):2301053.

[29] Zhu X, Wu K, Anderson SW, Zhang X. Wearable coaxially-shielded metamaterial for magnetic resonance imaging. Adv Mater. 2024;36(31):e2313692. 38569592 10.1002/adma.202313692

[30] Stoja E, Konstandin S, Philipp D, Wilke RN, Betancourt D, Bertuch T, Jenne J, Umathum R, Günther M. Improving magnetic resonance imaging with smart and thin metasurfaces. Sci Rep. 2021;11(1):16179. 34376748 10.1038/s41598-021-95420-w PMC8355254

[31] Wu K, Zhu X, Anderson SW, Zhang X. Wireless, customizable coaxially shielded coils for magnetic resonance imaging. Sci Adv. 2024;10(24):eadn5195. 38865448 10.1126/sciadv.adn5195 PMC11168459

[32] Wang B, Zhou J, Koschny T, Soukoulis CM. Nonlinear properties of split-ring resonators. Opt Express. 2008;16(20):16058–16063. 18825245 10.1364/oe.16.016058

[33] Lapine M, Shadrivov IV, Kivshar YS. Colloquium: Nonlinear metamaterials. Rev Mod Phys. 2014;86:1093–1123.

[34] Poutrina E, Huang D, Urzhumov Y, Smith DR. Nonlinear oscillator metamaterial model: Numerical and experimental verification. Opt Express. 2011;19:8312–8319. 21643082 10.1364/OE.19.008312

[35] Husu H, Siikanen R, Makitalo J, Lehtolahti J, Laukkanen J, Kuittinen M, Kauranen M. Metamaterials with tailored nonlinear optical response. Nano Lett. 2012;12(2):673–677. 22233139 10.1021/nl203524k

[36] Chen HT, Padilla WJ, Zide JM, Gossard AC, Taylor AJ, Averitt RD. Active terahertz metamaterial devices. Nature. 2006;444:597–600. 17136089 10.1038/nature05343

[37] Lee S, Kim S, Kim TT, Kim Y, Choi M, Lee SH, Kim JY, Min B. Reversibly stretchable and tunable terahertz metamaterials with wrinkled layouts. Adv Mater. 2012;24(26):3491–3497. 22688807 10.1002/adma.201200419

[38] Shadrivov IV, Kapitanova PV, Maslovski SI, Kivshar YS. Metamaterials controlled with light. Phys Rev Lett. 2012;109:Article 083902. 23002746 10.1103/PhysRevLett.109.083902

[39] Lewandowski W, Fruhnert M, Mieczkowski J, Rockstuhl C, Górecka E. Dynamically self-assembled silver nanoparticles as a thermally tunable metamaterial. Nat Commun. 2015;6:6590. 25779822 10.1038/ncomms7590

[40] Zhao X, Schalch J, Zhang J, Seren HR, Duan G, Averitt RD, Zhang X. Electromechanically tunable metasurface transmission waveplate at terahertz frequencies. Optica. 2018;5(3):303–310.

[41] Haus HA. *Waves and fields in optoelectronics.* Hoboken (NJ): Prentice-Hall; 1984.

[42] Kurs A, Karalis A, Moffatt R, Joannopoulos JD, Fisher P, Soljacic M. Wireless power transfer via strongly coupled magnetic resonances. Science. 2007;317(5834):83–86. 17556549 10.1126/science.1143254

[43] Brown RW, Cheng Y-CN, Haacke EM, Thompson MR, Venkatesan R. *Magnetic resonance imaging: Physical principles and sequence design.* John Wiley & Sons; 2014.

[44] Goerner FL, Clarke GD. Measuring signal-to-noise ratio in partially parallel imaging MRI. Med Phys. 2011;38(9):5049–5057. 21978049 10.1118/1.3618730 PMC3170395

[45] National Electrical Manufacturers Association, *Determination of signal-to-noise ratio (SNR) in diagnostic magnetic resonance imaging*. NEMA Standards Publication MS 1-2008 (R2014, R2020). Rosslyn (VA): National Electrical Manufacturers Association; 2021.

[46] Hayes CE, Axel L. Noise performance of surface coils for magnetic-resonance imaging at 1.5T. Med Phys. 1985;12(5):604–607. 4046995 10.1118/1.595682

