# Supplementary material for: Conformal Metamaterials with Active Tunability and Self-Adaptivity for Magnetic Resonance Imaging
Source: Research (Wash D C). 2024 Dec 23;7:0560. doi: 10.34133/research.0560 (PMC11665932; doi:10.34133/research.0560)
Supplement: Supplementary 1 — Texts S1 to S8 Figs. S1 to S14 Tables S1 to S3 [file research.0560.f1.pdf]

Supplementary Materials

Conformal Metamaterials with Active Tunability and Self-adaptivity for Magnetic Resonance Imaging

Ke Wu[1,3], Xia Zhu[1,3], Xiaoguang Zhao[2,3], Stephan W. Anderson[2,3]*, and Xin Zhang[1,3]*

[1]Department of Mechanical Engineering, Boston University, Boston, MA 02215, United States.
[2]Boston University Chobanian & Avedisian School of Medicine, Boston, MA, 02118, United States.
[3]Photonics Center, Boston University, Boston, MA 02215, USA.

*Address correspondence to: xinz@bu.edu (X.Z.); sande@bu.edu (S.W.A.)

This file includes:

Texts: S1 to S8

Figs: S1 to S14

Table: S1 to S3

Table of Contents

Supplementary Texts 3

Text S1. Fabrication of the metamaterial 3

Text S2. Selection of varactors for optimizing the metamaterial 3

Text S3. Mathematical modeling for the unit cell of the metamaterial 4

Text S4. Frequency detuning effect of the metamaterial 6

Text S5. Simulation results of the magnetic field distribution 7

Text S6. Discussion on SNR assessment of MRI scans 8

Text S7. Simulation model for SNR estimations 9

Text S8. MRI validation with the pineapple 9

Supplementary figures 10

Fig. S1. Fabrication process of metamaterial. 10

Fig. S2. Evaluation of varactors on SNR performance. 11

Fig. S3. Experimental setup for EM characterizations. 12

Fig. S4. A comparative schematic illustration of the proposed metamaterial and a conventional non-adaptive metamaterial working in an MRI system. 13

Fig. S5. Susceptibility of metamaterial resonance frequency. 14

Fig. S6.Experimental setup for magnetic field mapping. 15

Fig. S7. Simulated magnetic field distribution along the planar metamaterial cross-section. 16

Fig. S8. Simulated magnetic field distribution along the semi-cylindrical metamaterial cross-section. 17

Fig. S9. Two-image method for SNR evaluation. 18

Fig. S10. Comparison between different SNR evaluation methods. 19

Fig. S11. Numerical simulation models to evaluate SNR for planar (A) and semi-cylindrical (B) metamaterials. 20

Fig. S12. Experimental setup for MRI validations with porcine leg. 21

Fig. S13. MRI scans of ex vivo porcine leg by the BC with and without the metamaterial. 22

Fig. S14. MRI validation with pineapple 23

Supplementary Tables 24

Table S1. Components applied in the unit cells of metamaterial. 24

Table S2. Varactors employed for evaluating SNR performance. 24

Table S3.Parameters employed in mathematical derivation. 24

Supplementary Texts

Text S1. Fabrication of the metamaterial

To greatly eases design and process modification, we developed a fabrication process based on laser cutting and acid etching for the metamaterial from concept to prototype, as shown in Fig. S1. Firstly, we stacked an acrylic board, a flexible polyimide (PI) sheet, a copper foil, and a tape layer in sequence to form a multilayer structure, as shown in Fig. S1A, in which the acrylic board provides support for the following fabrication process, the PI serves as the substrate of the metamaterial, and the tape is employed as the mask layer to pattern the copper foil below. Next, the multilayer structure was placed in the laser cutting machine to pattern the tape, as shown in Fig. S1B. Next, the residue of the patterned tape was removed, exposing the area for the subsequent copper acid etching shown in Fig. S1C. Subsequently, the structure was moved to piranha solution, a mixture of sulfuric acid (H_2SO_4) and hydrogen peroxide (H_2O_2), to etching the exposed copper foil shown in Supplementary Fig. S1D. With the acid etching, the copper-based structure of the metamaterial is achieved, as show in Fig. S1E. Lastly, we solder the RLC and varactor components into the copper structure and release the supporting material to form the final structure of metamaterial; the fabrication result are depicted in Fig. S1F. The components employed in the metamaterial are listed in Table S1.

Text S2. Selection of varactors for optimizing the metamaterial

In the metamaterial design, many integrated components (capacitors, inductors, and varactors) were employed to realize the tunability and self-adaptivity. The employment of these electronic components unavoidably introduces series resistance, leading to inherent losses and a reduction in the Q value of the metamaterial. In order to achieve the optimal performance of metamaterial, for the capacitors and inductors, we meticulously selected the ones with high Q value and non-magnetic terminations, which are listed in Table S1. In the selection of varactors, we faced a dual requirement: the inner resistance should be minimized, while the capacitance tuning range must remain within an appropriate range to preserve the desired characteristics of the metamaterial, i.e., tunability and self-adaptivity. To explore the impact of inner resistance on SNR enhancement, we deliberately chose three varactors with varying inner resistance values, ranging from low to high. For the purpose of assessing SNR performance, we employed varactors SMV1405, SMV2020, and SMV1247, considering their resistance and capacitance tuning range. These varactors were selected to achieve frequency tunability, and their impact on SNR was evaluated. Additionally, we used varactors SMV1413, SMV1405, and SMV1247

for achieving self-adaptivity of metamaterials. With these varactors, the fabricated metamaterial in a 2 × 2 array fashion is depicted in Fig. S2A. Detailed information about these varactors can be found in Table S2. To experimentally assess the influence of different varactors, we conducted scans of a bottle-shaped phantom filled with mineral oil in the presence of metamaterials equipped with various sets of varactors. Employing consistent scanning parameters and imaging sequences, we generated corresponding SNR images presented in Supplementary Figs. S2B to F. To begin, we extracted and plotted the metamaterial-enhanced SNR value along the dashed lines in the images (depicted in Supplementary Figs. S2B, C and D) to evaluate the impact of varactors for frequency tunability on SNR performance. The maximum SNR values obtained for these three images were 1618.1, 1274.3, and 678.2, corresponding to varactor's inner resistances of 0.8, 2.5, and 4.9 Ω, respectively. Based on these experimental results, varactor SMV1405 was selected for achieving frequency tunability due to its combination of low inner resistance and an appropriate tuning range of capacitance. Furthermore, we extracted and plotted the metamaterial-enhanced SNR along the dashed lines in the images (illustrated in Figs. S2B, E, and F) to assess the varactors for self-adaptivity. For varactor's inner resistances of 0.3, 0.8, and 4.9Ω, the corresponding maximum SNR values were 1618.1, 1279.5, and 409, respectively. It's worth noting that not only the inner resistance impacts SNR performance, but also the capacitance of the varactor influences SNR enhancement due to different coupling coefficients between VLRR and CCLSR within each unit cell. Building upon these experimental findings, we ultimately selected varactor SMV1413 to enable self-adaptivity within the metamaterial.

Text S3. Mathematical modeling for the unit cell of the metamaterial

The resonance response of the unit cell of the metamaterial may be derived based on the coupled mode theory (CMT), which is widely employed to describe the response of electromagnetic systems.

$$\frac{da_1}{dt} = \left[j\omega_1(V_t) - \frac{1}{\tau_{e1}} - \frac{1}{\tau_{o1}}\right] a_1 + jka_2 + \sqrt{\frac{2}{\tau_{e1}}} s_+ \qquad (S1)$$

$$\frac{da_2}{dt} = \left[j\omega_2(|a_2|) - \frac{1}{\tau_{e2}} - \frac{1}{\tau_{o2}}\right] a_2 + jka_1 + \sqrt{\frac{2}{\tau_{e2}}} s_+ \qquad (S2)$$

where a_1 and a_2 is the mode amplitude of the CCLSR and VLSR. ω_1 and ω_2 are the resonance frequency of the CCLSR and VLSR, correspondingly. $(1/\tau_{e1}+1/\tau_{o1})$ and $(1/\tau_{e2}+1/\tau_{o2})$ are the

decay rate of the oscillating strength due to radiation and intrinsic losses of the CCLSR and VLSR, respectively. k is the coupling factor between these two resonators and s_+ is the excitation signal, which is a harmonic function with frequency ω (i.e., $s_+=|s_+|e^{j\omega t}$) when considering the MRI application scenario. By converting the time domain differential equations to the frequency domain, the oscillating amplitude of the two resonators may be calculated by numerically solving the matrix equations:

$$j\omega\begin{bmatrix}a_1\\a_2\end{bmatrix}=j\begin{bmatrix}\omega_1(V_t)+j\left(\frac{1}{\tau_{e1}}+\frac{1}{\tau_{o1}}\right) & k\\ k & \omega_2(|a_2|)+j\left(\frac{1}{\tau_{e2}}+\frac{1}{\tau_{o2}}\right)\end{bmatrix}\begin{bmatrix}a_1\\a_2\end{bmatrix}+\begin{bmatrix}\sqrt{\frac{2}{\tau_{e1}}}\\\sqrt{\frac{2}{\tau_{e2}}}\end{bmatrix}s_+ \qquad (S3)$$

In order to solve Eq. S3, the expressions for ω_1 and ω_2 need to be determined. Firstly, the effective capacitance of the CCLSR may be expressed by:

$$C_{CCLSR}=C_s+\frac{1}{\frac{1}{C_1+C_{var1}(V_t)}+\frac{1}{C_2}+\frac{1}{C_3}}=C_s+\frac{1}{\frac{1}{C_1+C_0\left(1+\frac{V_t}{V_P}\right)^{-M}+C_P}+\frac{1}{C_2}+\frac{1}{C_3}}$$
$$\approx C_s+C_1+C_0\left(1+\frac{V_t}{V_P}\right)^{-M}+C_P \qquad (S4)$$

The effective capacitance of the VLSR may be expressed by:

$$C_{VLRR}=C_{var2}(V_D)=C_0\left(1-\frac{V_D}{V_P}\right)^{-M}\approx C_0\left(1+\frac{MV_D}{V_P}\right) \qquad (S5)$$

As the main text states, ω_1 is controlled by the biasing voltage V_t, which may be expressed as:

$$\omega_1=\frac{1}{\sqrt{L_{CCLSR}C_{CCLSR}}}=\frac{1}{\sqrt{L_{CCLSR}\left(C_s+C_1+C_0\left(1+\frac{V_t}{V_P}\right)^{-M}+C_P\right)}}$$
$$=\omega_{10}\left(\frac{C_s+C_1+C_0\left(1+\frac{V_t}{V_P}\right)^{-M}+C_P}{C_s+C_1+C_P+C_0}\right)^{-\frac{1}{2}}=\lambda_1(V_t)\omega_{10} \qquad (S6)$$

in which L_{CCLSR} is the inductance of the 2-turn spiral coil in the CCLSR, C_S is the distributed capacitance of the spiral coil, C_0 is the initial capacitance of the varactor C_{var1}, C_P is the package

capacitance, M is a fitting exponent, and V_P is the intrinsic potential of the varactor obtained from the data sheet. ω_{10} is the resonance frequency of the CCLSR when its biasing voltage is 0 V. As for ω_2, depending on the oscillating strength of the VLSR, this may be expressed as:

$$\omega_2 = \frac{1}{\sqrt{L_{VLSR}C_{VLSR}}} \approx \frac{1}{\sqrt{L_{VLSR}C_0\left(1+\frac{MV_D}{V_P}\right)}} = (1-\lambda_2|a_2|)\omega_{20} \qquad (S7)$$

By solving for the solutions of a_1 and a_2 from Supplementary Equation (S3), we obtain:

$$a_2 = \frac{\left\{jk\sqrt{\frac{2}{\tau_{e1}}} + \left[j(\omega - \lambda_1(V_t)\omega_{10}) + \frac{1}{\tau_{e1}} + \frac{1}{\tau_{o1}}\right]\sqrt{\frac{2}{\tau_{e2}}}\right\}|s_+|}{\left[j\big(\omega - \omega_1(V_t)\big) + \frac{1}{\tau_{e1}} + \frac{1}{\tau_{o1}}\right]\left[j\big(\omega - \omega_2(|a_2|)\big) + \frac{1}{\tau_{e2}} + \frac{1}{\tau_{o2}}\right] + k^2} \qquad (S8)$$

$$a_1 = \frac{jka_2 + \sqrt{\frac{2}{\tau_{e1}}}|s_+|}{j(\omega - \lambda_1(V_t)\omega_{10}) + \frac{1}{\tau_{e1}} + \frac{1}{\tau_{o1}}} \qquad (S9)$$

With the solutions of a_1 and a_2, the spectral reflectance of the unit cell may be expressed as:

$$r = -1 + \frac{\sqrt{\frac{2}{\tau_{e1}}}a_1 + \sqrt{\frac{2}{\tau_{e2}}}a_2}{2|s_+|} \qquad (S10)$$

All of the parameters employed in the derivation process are listed in Table S3. The theoretical results are in close agreement with the measured results using these parameters.

Text S4. Frequency detuning effect of the metamaterial

In order to investigate the detuning effect of the metamaterial resonance frequency, and also demonstrate that the frequency tuning range is sufficient to compensate for the frequency deviations, we tested the resonance frequency shift in the presence of phantoms of differing composition, and also investigated the frequency shift when the metamaterial was configured in different shapes. When a phantom with a relative high permittivity is in close proximity to the metamaterial, the effective capacitance of metamaterial becomes larger, leading to a resonance frequency shift to lower values. Three home-made, bottle-shaped phantoms containing air, mineral oil (NO-TOX® Food Grade Oil, Bel-Ray), and water with the relative permittivity of ~1, ~2.1, and ~80 were prepared for the semi-cylindrical metamaterial. The typical relative permittivity of the human body approximates 40~60, such the permittivity range

of the employed phantoms is wide enough to mirror true clinical MRI applications. Using the same experimental setup for the characterization of frequency tunability, we initially tuned the metamaterial to a resonant frequency of 127.7 MHz in the absence of a phantom by setting the biasing voltage to 0.51 V, and then measured the spectral reflectance when keeping the biasing voltage constant. The spectra are depicted in Fig. S5A. Since the permittivity of mineral oil is close to air, there is no obvious frequency detuning effect in the presence of the oil phantom. When in proximity to the water phantom, the metamaterial resonance frequency shifted to 127 MHz, which could be tuned back to 127.7 MHz by increasing the biasing voltage from 0.51 to 0.59 V. Of note, in the case of the flexible metamaterial reported herein, the deformation also exerts an influence on its resonance frequency by altering the coupling coefficient between unit cells in the metamaterial. As a result, we measured the reflection spectra when the metamaterial was in a planar configuration, as well as curved configurations with arc angle of 135° and 180°. Initially, the biasing voltage was adjusted to 0.66V to tune the planar metamaterial resonating at 127.7 MHz. With the same biasing voltage, the metamaterial resonates at 128.4 MHz, and 128.9 MHz for arc angles of 135° and 180°, respectively (Fig. S5B), which could be modified to an optimal value of 127.7 MHz by adjusting the biasing voltage from 0.66 to 0.58 V and 0.51 V, respectively. The test results demonstrate the frequency tuning range is sufficient to compensate for the detuning effects of simulated differences in subject body composition during imaging as well as changes due to variations in configurations and, thereby, realize an optimized frequency match between the metamaterials and the MRI system.

Text S5. Simulation results of the magnetic field distribution

In the main text, we have experimentally depicted the magnetic field distribution in the vicinity of the metamaterial. Besides the experimental results, we also built a numerical simulation model in CST to investigate the magnitude and phase information of the magnetic field. As SNR enhancement by the metamaterial is a result of its capability to confine and enhance the resonant electromagnetic field in the MRI system, we focused on simulating the magnetic field during the on-resonance state to mimic this reception phase. Additionally, we employed the off-resonance condition to reflect the transmission phase in the MRI system. While conducting the numerical simulations, we encountered the challenge of modeling a dynamic nonlinear response accurately. To estimate the field distribution under high incident power conditions, we used the static response by assuming an increase in capacitance of the varactor and the induced decrease of the resonance frequency. To maintain consistency with our experimental setups, we conducted simulations of the magnetic field on the same cutting

planes employed in our experiments. Under low and high power excitation, the simulated magnitudes of the magnetic field are depicted in Figs. S7A and B, respectively. And the magnitude of the field strength along the white dashed lines in Figs. S7A and B were extracted and plotted in Fig. S7E. Remarkably, the pattern of field magnitude closely aligns with the experimental results depicted in Figs. 2A and B in the main text, providing further validation of our near-field mapping results. In addition to plotting the magnitude of the magnetic field, we also investigated the phase information of the magnetic field under both low and high excitation power conditions, which are presented in Figs. S7C and D, respectively. Moreover, we extracted the angles of the magnetic field's phase along the white dashed lines in Figs. S7C and D and plotted them in Fig. S7F. The phase information of the magnetic field in the area far away from the metamaterial demonstrates that there is no such a phase transition region where the original magnetic field and the induced magnetic field reverse cancel each other, avoiding the RF artifacts in the imaging area due to magnetic field cancellation. Furthermore, we conducted similar simulations for the case where the metamaterial is configured in a semi-cylindrical shape. The simulation results are depicted in Fig. S8, reaffirming that the same conclusions can be drawn as those observed with the planar metamaterial.

Text S6. Discussion on SNR assessment of MRI scans

In this study, we employed a two-image method, recommended as NEMA Method 2 (N2) by the National Electrical Manufacturers Association (NEMA). In this approach, a noise image is acquired with the phantom in its original position, but without RF excitation. To validate the feasibility of this method in evaluating the metamaterial's performance in enhancing SNR, we also applied two other SNR evaluation standards, NEMA Method 1 (N1) and NEMA Method 4 (N4), as outlined in the same NEMA guidelines. The N1 method calculates SNR from a difference image of two repeated (identical) acquisitions during the same imaging session with minimal time interval. The N4 method involves acquiring a single image and measuring noise in a signal-free region (e.g., air), valued for its simplicity and speed. Using these three methods, we conducted a series of phantom scans, acquiring SNR images both with and without the metamaterial. The resulting SNR images are shown in Figs. S10 (A), (B), and (C) for the N1, N2, and N4 methods, respectively, all using a common scale bar for SNR values. We also calculated the SNR enhancement ratio by normalizing the SNR values from the metamaterial-enhanced images to those without the metamaterial across the three methods. The SNR images from these methods show strong alignment, with the SNR enhancement ratios demonstrating particularly high agreement. This consistency across the three methods further

supports the feasibility of our approach based on N2 for SNR assessment and strengthens the conclusions of the manuscript.

Text S7. Simulation model for SNR estimations

In order to accurately evaluate the SNR through the numerical simulation method, a high-pass birdcage coil was initially built with the same geometry as the Philips 3T MRI scanner. The birdcage coil could generate a homogeneous circularly polarized magnetic field mimicking the B_1 field in the actual MRI. The mineral oil phantom was modeled by a cylindrically shaped material with a relative permittivity of 2.1, electric conductivity of 0.175 S/m, and a material density of 800 kg/m^3. For the simulation model of the metamaterial, the configurations and dimensions are the same as the fabricated sample described above. The modeled phantom was placed along the top surface of the metamaterial, with the separation distance between the top surface of the metamaterial and the bottom surface of phantom approximating 20 mm. The simulation models for the metamaterial presenting in planar and semi-cylindrical configurations are depicted in Figs. 10A and B, respectively.

Text S8. MRI validation with the pineapple

In addition to the experimental MRI validation using the mineral oil phantom and ex vivo porcine leg, we also imaged a pineapple as a larger biological sample to demonstrate the performance of the metamaterial when configured into an arc shape with an arc angle smaller than 180°, as shown in Fig. S14A. Gradient echo imaging (GRE) was employed, using a repetition time (TR) and echo time (TE) of 100 and 4.6 ms, respectively. The pixel size was 1 × 1 mm, the slice thickness was 5 mm, and the matrix size was 256 × 256 mm for images on sagittal cutting planes. As shown in Figs. S14B and C, when compared to images acquired using the BC only, images with enhanced SNR were achieved in the presence of the metamaterial. The SNR enhancement of these acquired images of the pineapple further provide a preliminary demonstration of the potential of this technology applied in MRI.

Supplementary figures

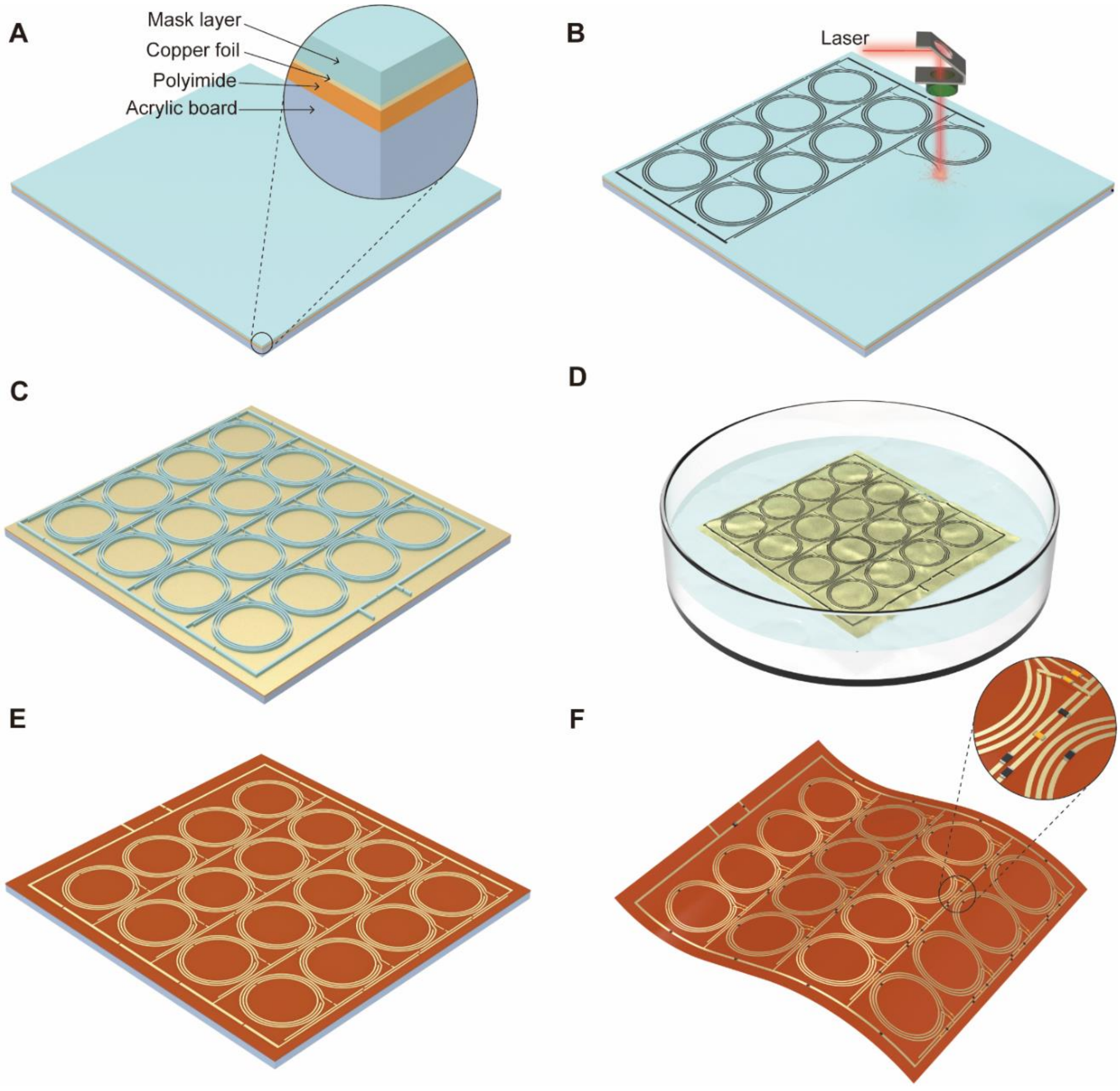

Fig. S1. Fabrication process of metamaterial. (**A**) Stacked structures for fabricating metamaterial. (**B**) Laser cutting for patterning the mask layer. (**C**) Remove residues of patterned tape to expose the copper foil to be removed. (**D**) Acid etching of copper. (**E**) Acid etching result. (**F**) Solder components and release the supporting acrylic board.

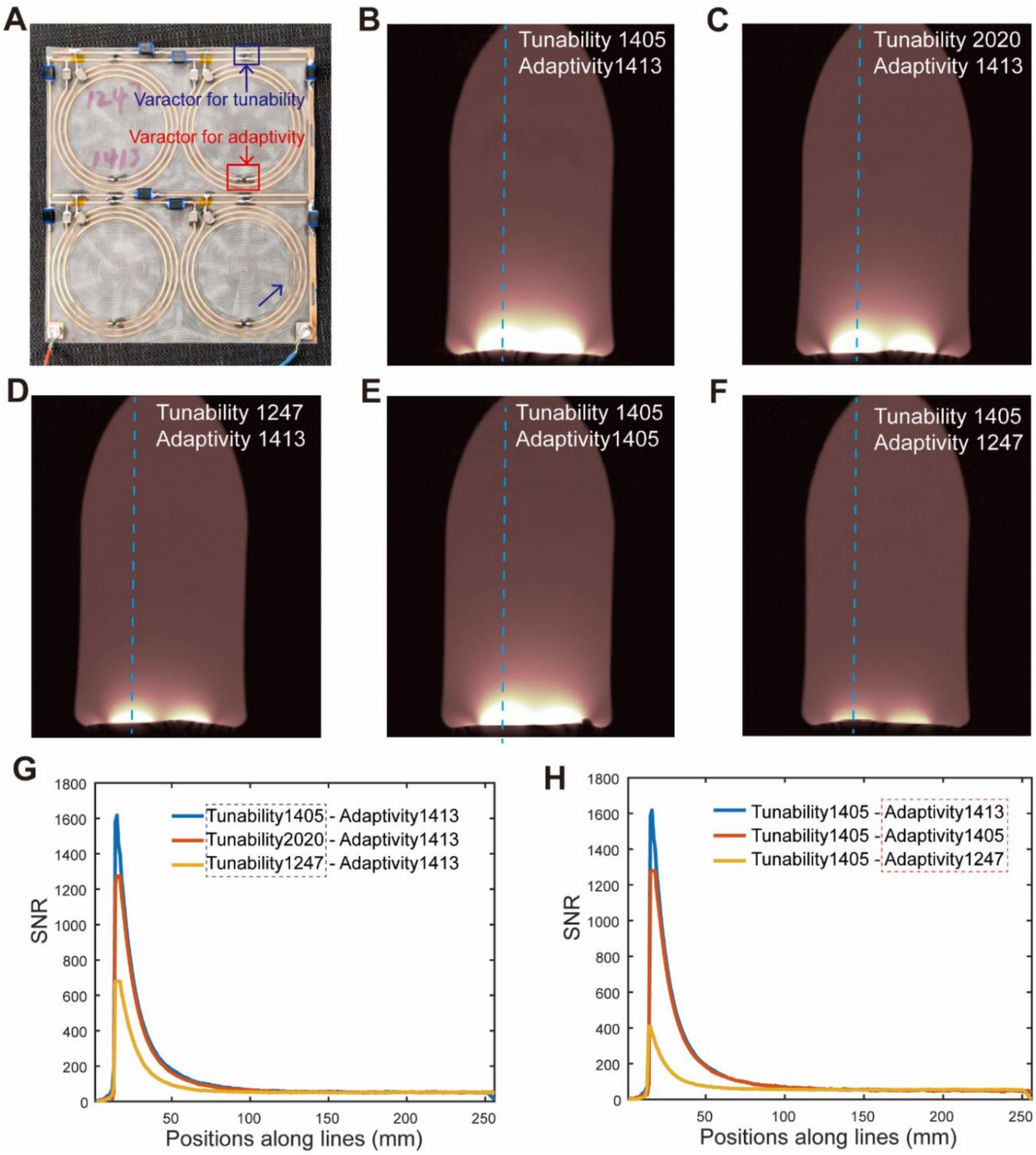

Fig. S2. Evaluation of varactors on SNR performance. (**A**) Metamaterial in a 2 × 2 array fashion. (**B** to F) metamaterial-enhanced SNR images with different sets of varactors, as indicated by the legends in the corresponding figures. (**G**) Comparison of SNR values along blue dashed lines in (B), (C), and (D) for evaluating the varactors for realizing frequency tunability. (**H**) Comparison of SNR values along blue dashed lines in (B), (E), and (F) for evaluating the varactors for realizing self-adaptivity

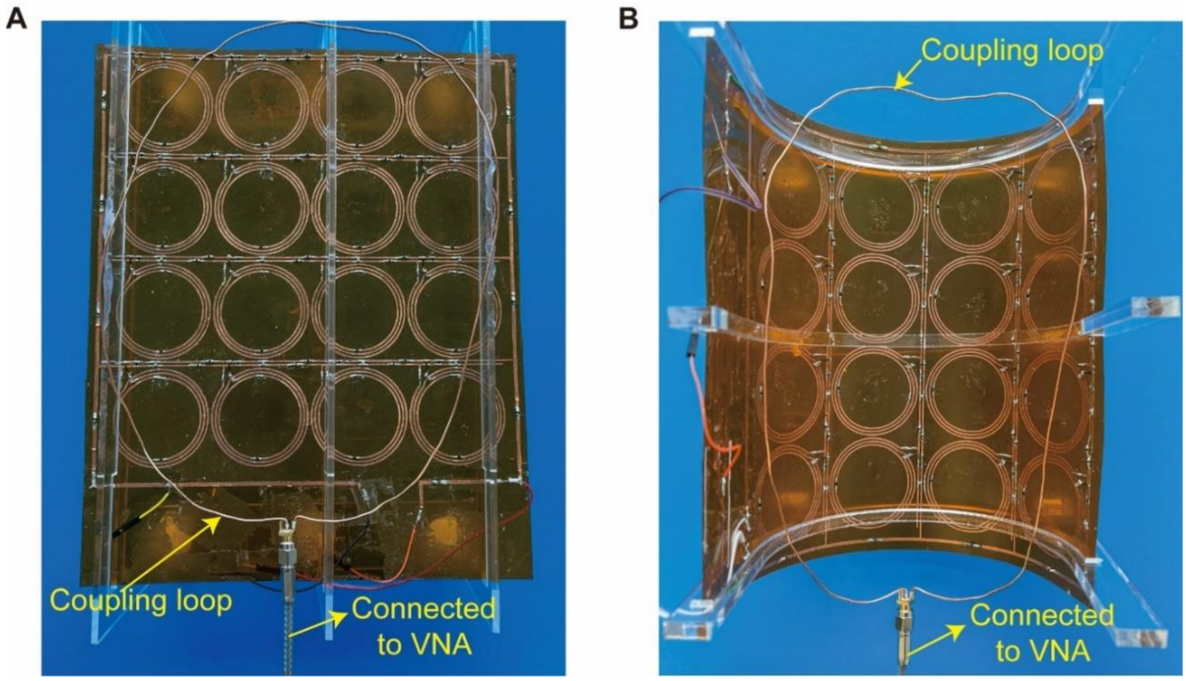

Fig. S3. Experimental setup for EM characterizations. (**A**) Characterization setup for the planar metamaterial. (**B**) Characterization setup for the semi-cylindrical metamaterial.

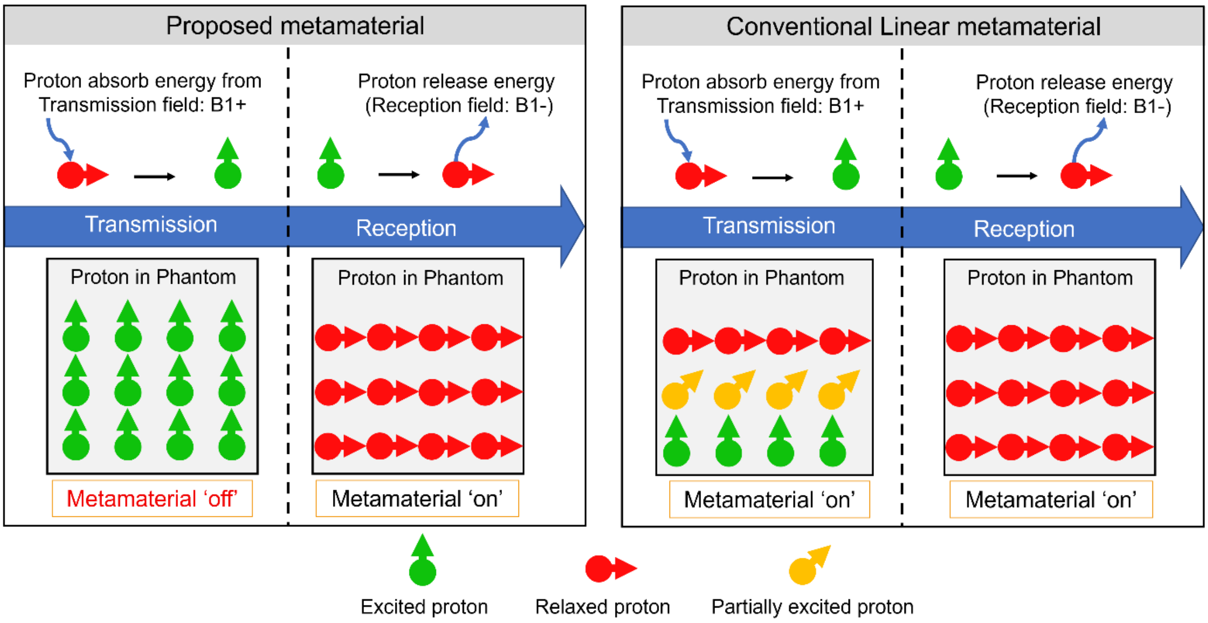

Fig. S4. A comparative schematic illustration of the proposed metamaterial and a conventional non-adaptive metamaterial working in an MRI system.

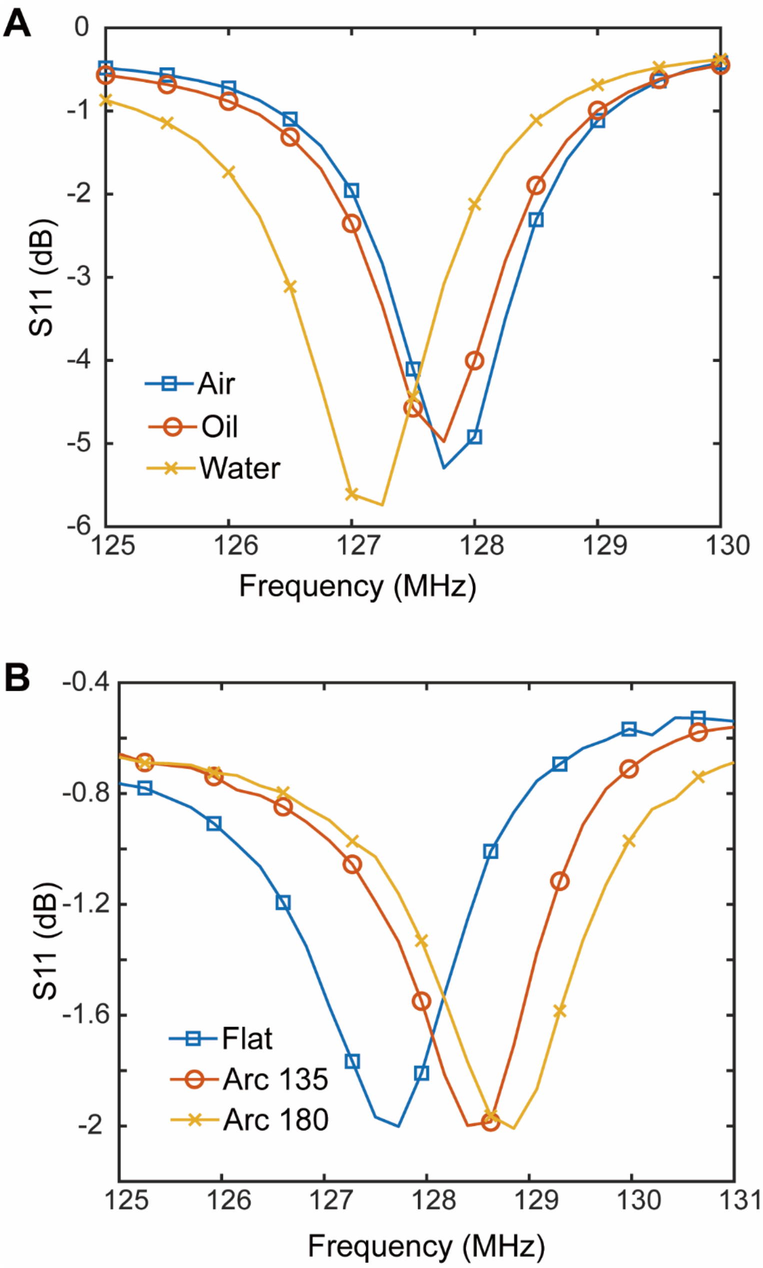

Fig. S5. Susceptibility of metamaterial resonance frequency. (**A**) Frequency detuning due to different phantoms. (**B**) Frequency detuning due to deformation.

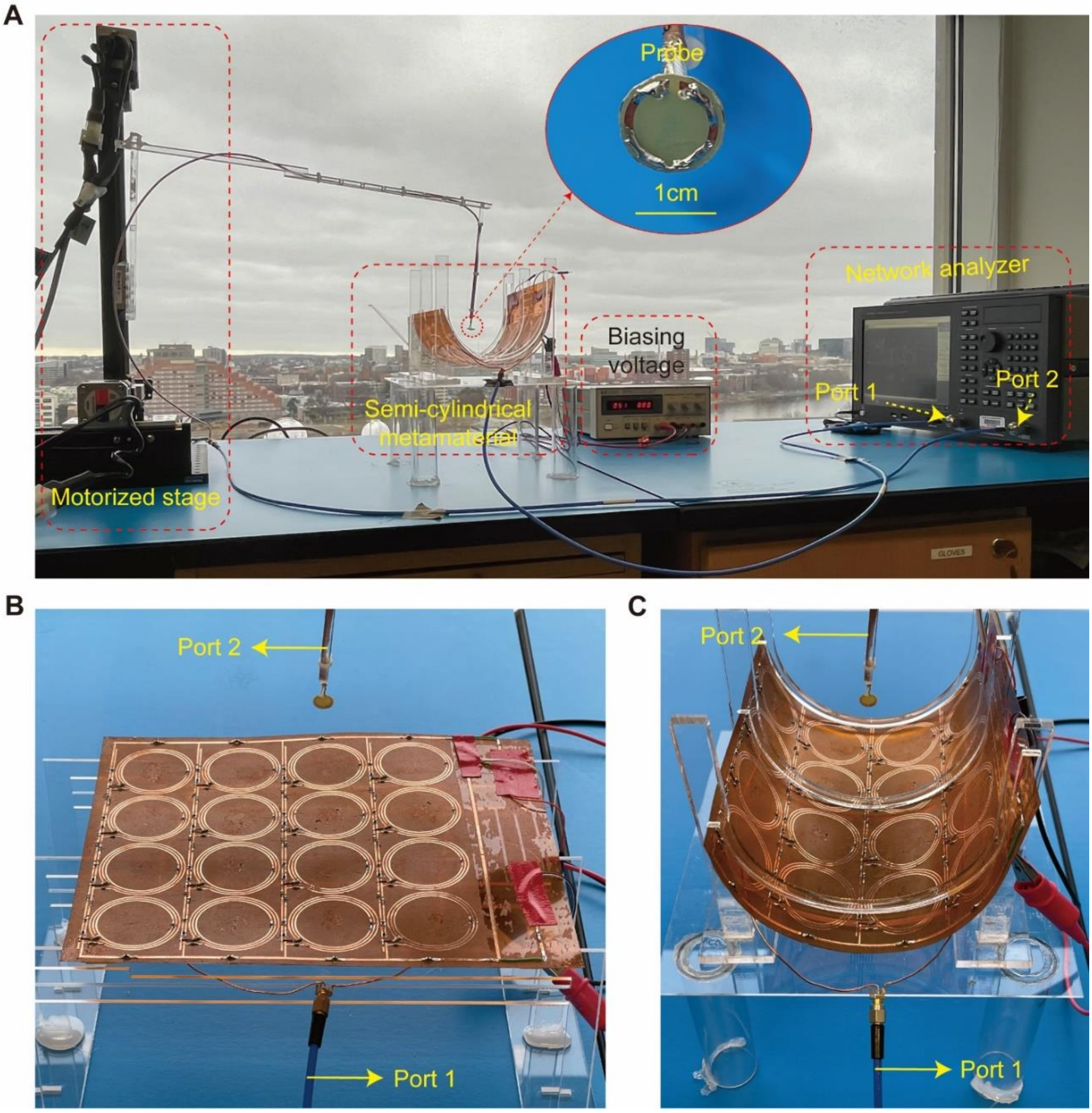

Fig. S6.Experimental setup for magnetic field mapping. (**A**) Overview of the bench test system for characterizing metamaterial. (**B**) Magnified view of the setup for the planar metamaterial. (**C**) Magnified view of the setup for the semi-cylindrical metamaterial.

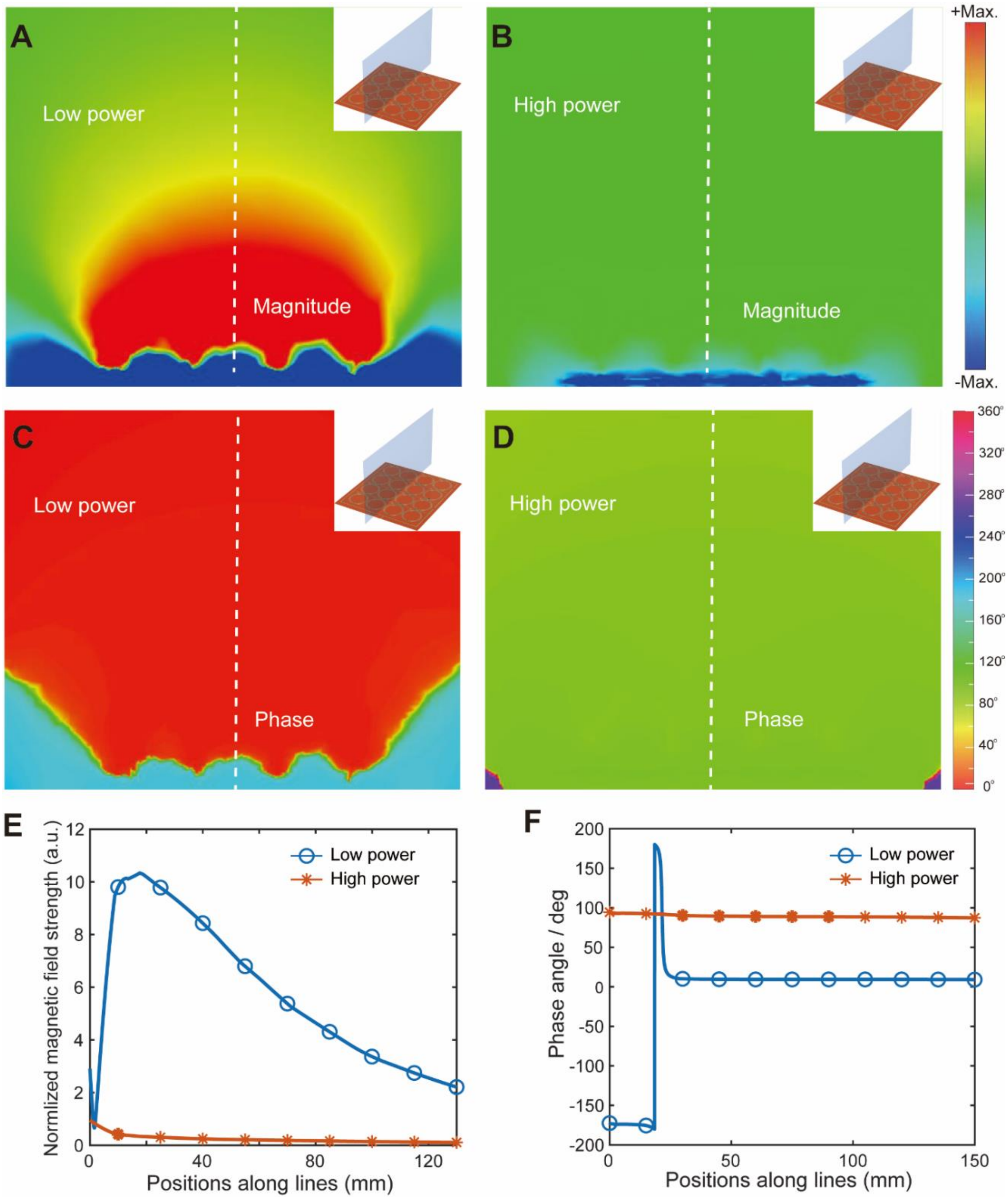

Fig. S7. Simulated magnetic field distribution along the planar metamaterial cross-section. (**A** and **C**) Magnitude and phase information of the magnetic field strength distributed along the planar metamaterial cross-section. (**B** and **D**), Magnitude and phase information of the magnetic field distributed along the semi-cylindrical metamaterial cross-section. (**E**) Magnitude of magnetic field strength along white dashed lines in (A) and (B). (**F**) Phase of the magnetic field along white dashed lines in (C) and (D).

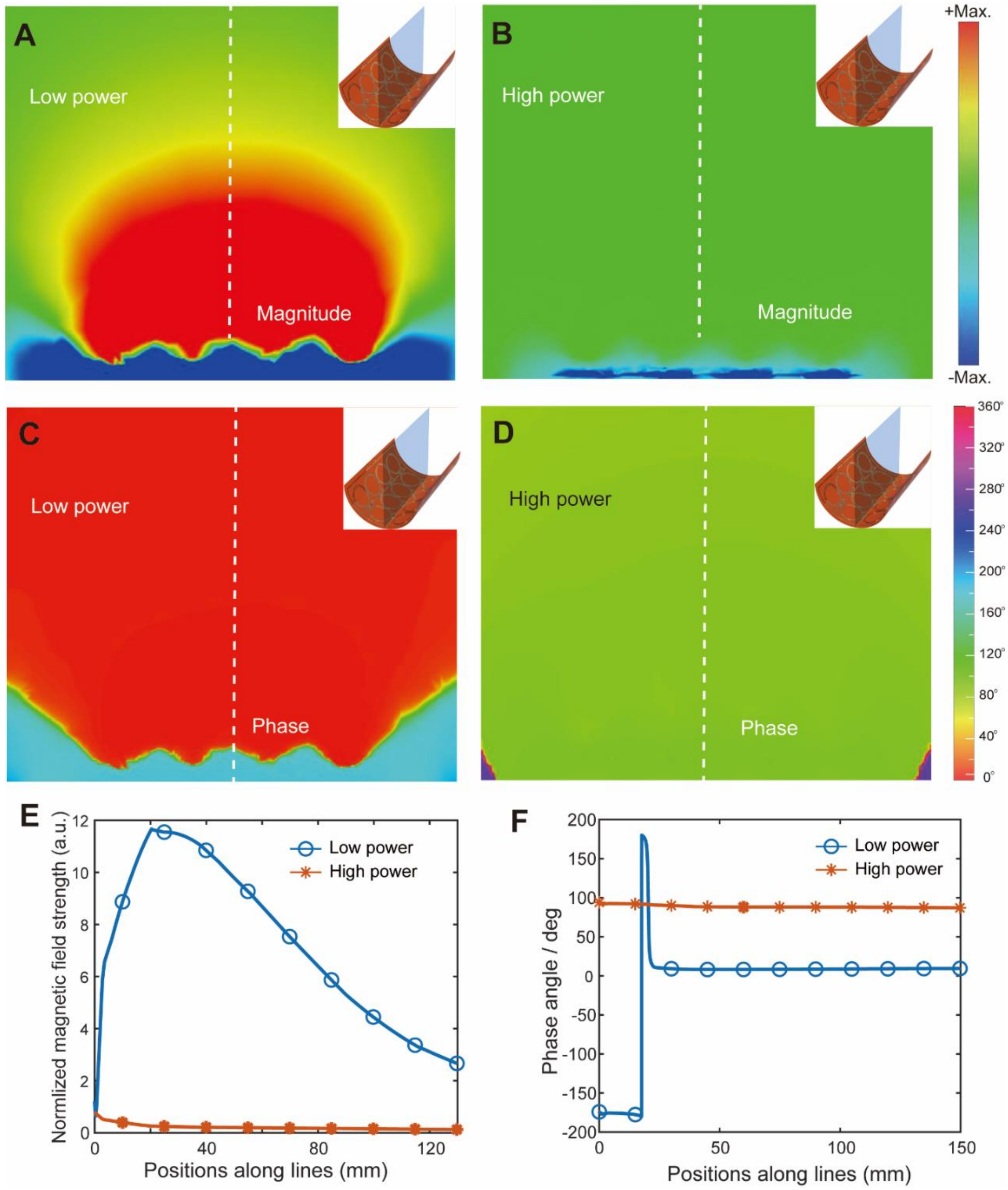

Fig. S8. Simulated magnetic field distribution along the semi-cylindrical metamaterial cross-section. (**A** and **C**) Magnitude and phase information of the magnetic field strength distributed along the planar metamaterial cross-section. (**B** and **D**), Magnitude and phase information of the magnetic field distributed along the semi-cylindrical metamaterial cross-section. (**E**) Magnitude of magnetic field strength along white dashed lines in (A) and (B). (**F**) Phase of the magnetic field along white dashed lines in (C) and (D).

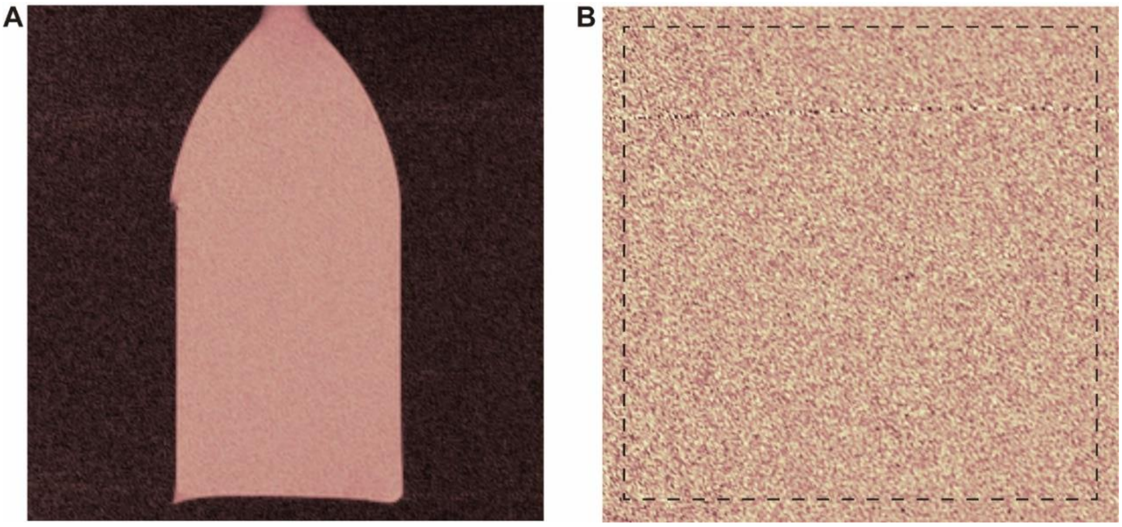

Fig. S9. Two-image method for SNR evaluation. (**A**) MRI image of the phantom, used to measure the signal. (**B**) Image capture with the transmission amplifier off, the standard deviation of which (dotted black frame) was employed to derive image noise.

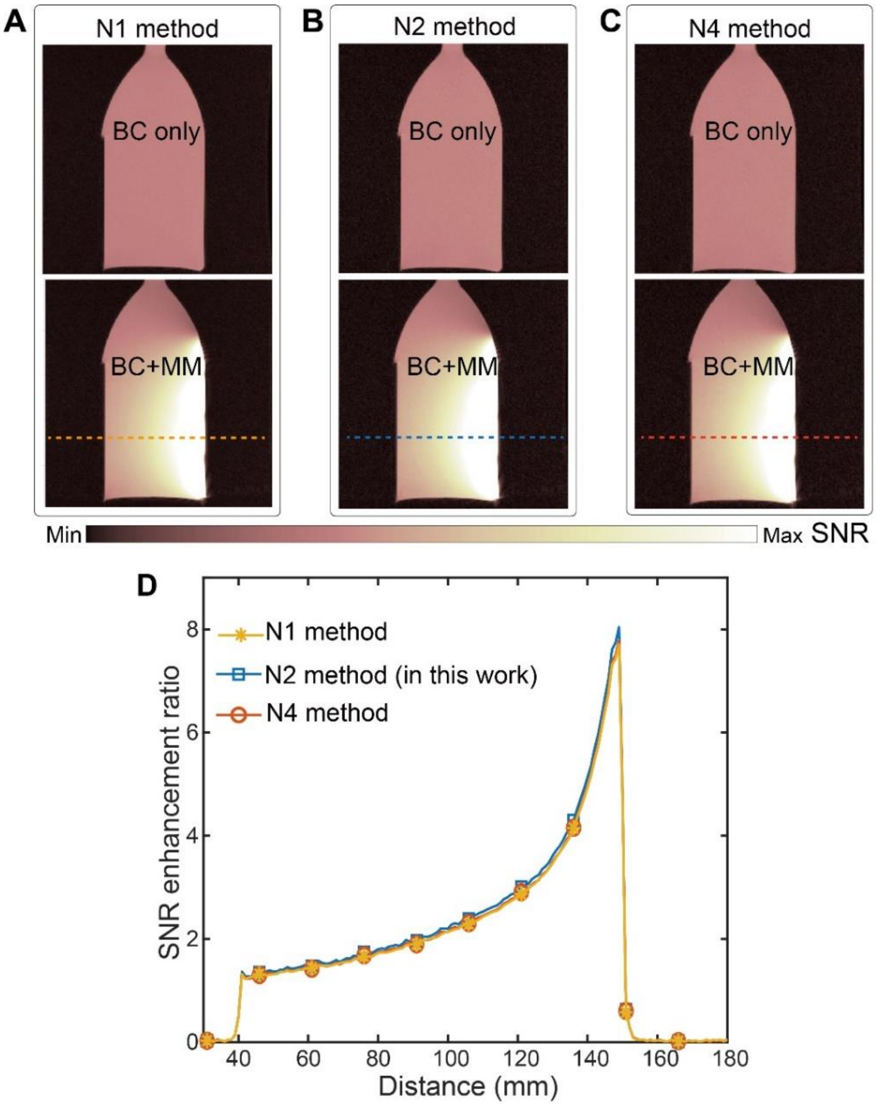

Fig. S10. Comparison between different SNR evaluation methods. (**A**) SNR images with and without metamaterial based on SNR evaluation method N1.(**B**) SNR images based on N2 adopted in the manuscript (**C**) SNR images based on N4. (**D**) SNR enhancement ratios along dashed lines in (A to C) based on different methods.

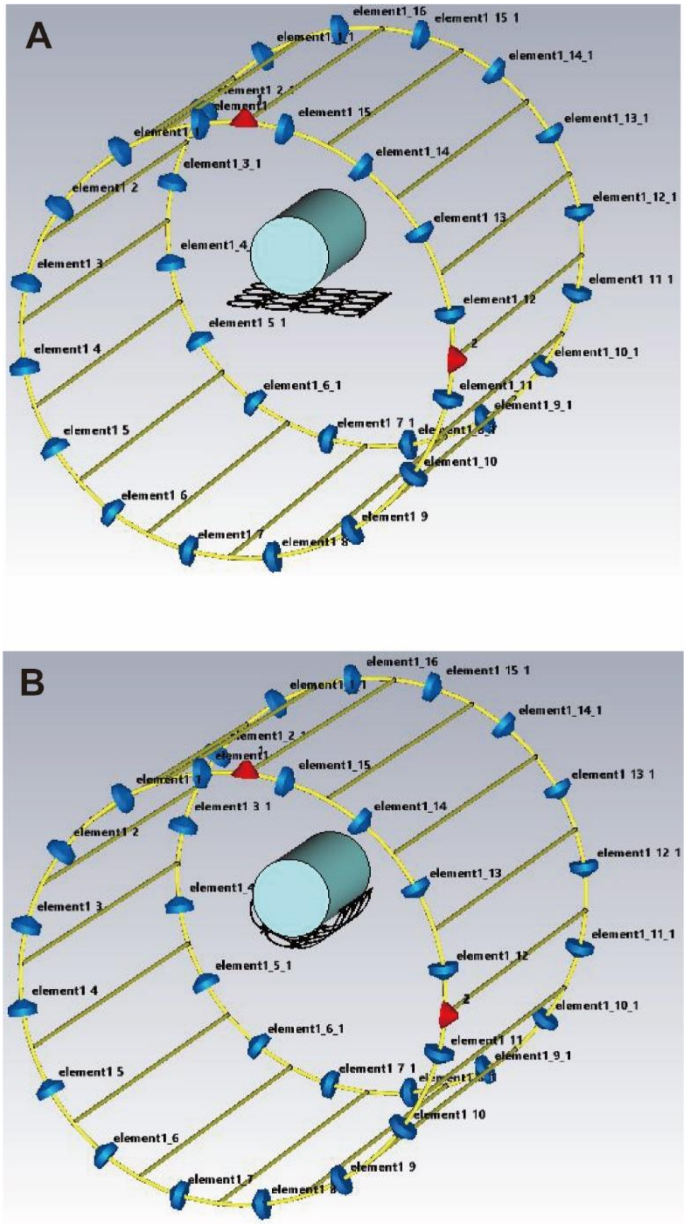

Fig. S11. Numerical simulation models to evaluate SNR for planar (A) and semi-cylindrical (B) metamaterials.

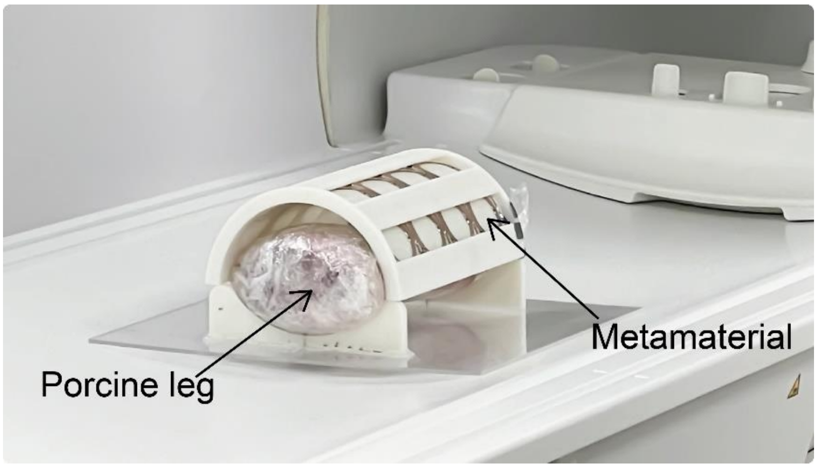

Fig. S12. Experimental setup for MRI validations with porcine leg.

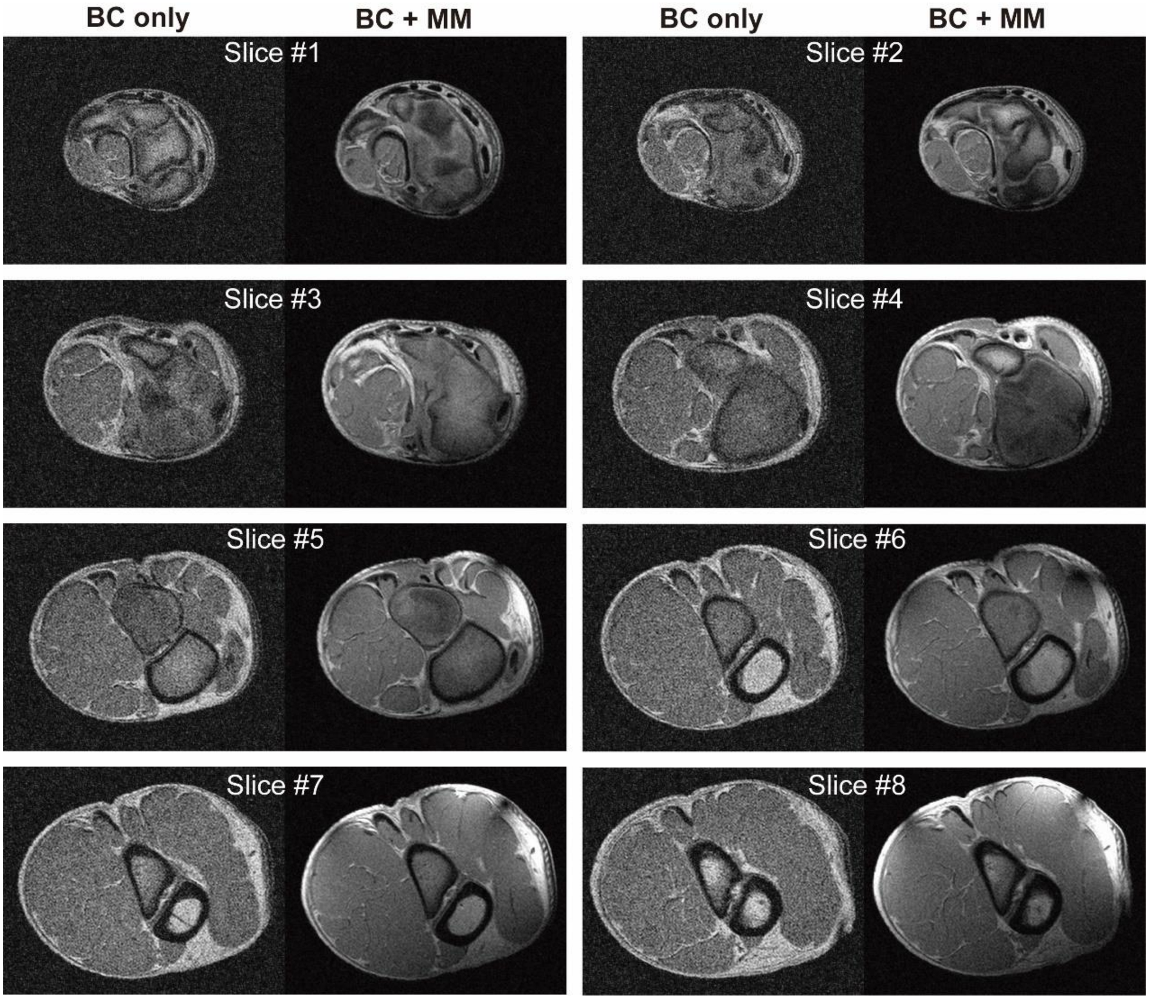

Fig. S13. MRI scans of ex vivo porcine leg by the BC with and without the metamaterial.

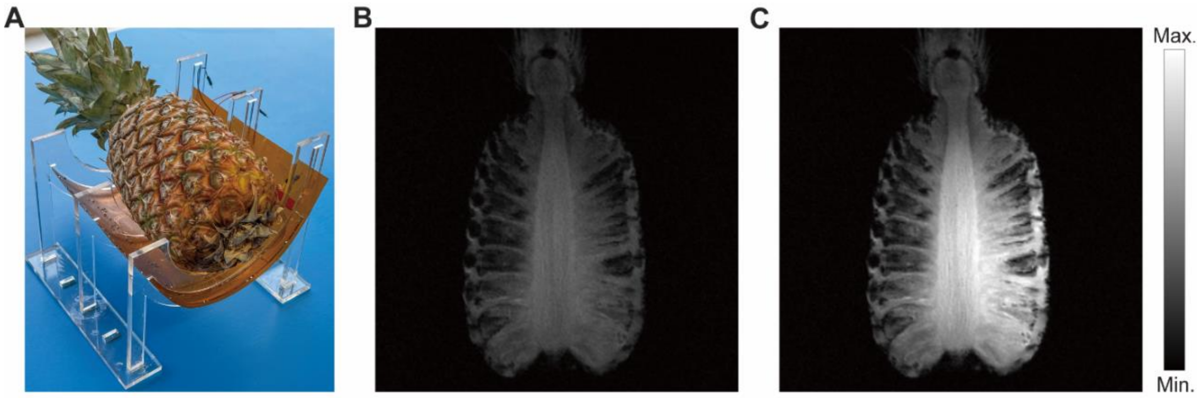

Fig. S14. MRI validation with pineapple. (**A**) Experimental setup in the MRI system. (**B**) Image of the pineapple in the sagittal plane captured by the BC only in the absence of metamaterial, employed as a reference. (**C**) Image captured by the body coil in the presence of the metamaterial. All images are normalized to the same scale bars.

Supplementary Tables

Table S1. Components applied in the unit cells of metamaterial.

Component	Description	Company	Part No.
L_1	Inductor, 33μH	Coilcraft	1812CS-333XJLC
L_2	Inductor, 33μH	Coilcraft	1812CS-333XJLC
C_1	Capacitor, 0pF	-	-
C_2	Capacitor, 100pF	Knowles Syfer	111121K00101GQTAF9LM
C_3	Capacitor, 100pF	Knowles Syfer	111121K00101GQTAF9LM
C_{var1}	Varactor, 0.63~2.67 pF	Skyworks	SMV1405
C_{var2}	Varactor, 1.77~9.24 pF	Skyworks	SMV1413

Table S2. Varactors employed for evaluating SNR performance.

Varactor	Capacitance	Tuning voltage	Inner resistance
SMV 1413	9.24~1.77 pF	0~30 V	0.3 Ω
SMV 1405	2.67~0.63 pF	0~30 V	0.8 Ω
SMV 2020	3.2~0.35 pF	0~20 V	2.5 Ω
SMV 1247	8.86~0.64 pF	0~8 V	4.9 Ω

Table S3.Parameters employed in mathematical derivation.

Component	Value	Component	Value
C_S	0.5 pF	V_P	0.77 V
L_{CCLSR}	0.64 μH	C_0	2.37 pF
M	0.5	k	0.5 / ω_{10}
C_P	0.29 pF	L_{VLSR}	0.24 μH
τ_{01}	90 / ω_{10}	τ_{e1}	80 / ω_{10}
τ_{02}	200 / ω_{20}	τ_{e2}	3200 / ω_{20}
